# Acylated-acyl carrier protein stabilizes the *Pseudomonas aeruginosa* WaaP lipopolysaccharide heptose kinase

**DOI:** 10.1038/s41598-018-32379-1

**Published:** 2018-09-20

**Authors:** Naomi N. K. Kreamer, Rajiv Chopra, Ruth E. Caughlan, Doriano Fabbro, Eric Fang, Patricia Gee, Ian Hunt, Min Li, Barbara C. Leon, Lionel Muller, Brian Vash, Angela L. Woods, Travis Stams, Charles R. Dean, Tsuyoshi Uehara

**Affiliations:** 1 Infectious Diseases, Novartis Institutes for Biomedical Research, Emeryville, CA USA; 2 Chemical Biology and Therapeutics, Novartis Institutes for Biomedical Research, Cambridge, MA USA; 3 Chemical Biology and Therapeutics, Novartis Institutes for Biomedical Research, Basel, Switzerland

## Abstract

Phosphorylation of *Pseudomonas aeruginosa* lipopolysaccharide (LPS) is important for maintaining outer membrane integrity and intrinsic antibiotic resistance. We solved the crystal structure of the LPS heptose kinase WaaP, which is essential for growth of *P*. *aeruginosa*. WaaP was structurally similar to eukaryotic protein kinases and, intriguingly, was complexed with acylated-acyl carrier protein (acyl-ACP). WaaP produced by *in vitro* transcription-translation was insoluble unless acyl-ACP was present. WaaP variants designed to perturb the acyl-ACP interaction were less stable in cells and exhibited reduced kinase function. Mass spectrometry identified myristyl-ACP as the likely physiological binding partner for WaaP in *P*. *aeruginosa*. Together, these results demonstrate that acyl-ACP is required for WaaP protein solubility and kinase function. To the best of our knowledge, this is the first report describing acyl-ACP in the role of a cofactor necessary for the production and stability of a protein partner.

## Introduction

Bacterial resistance to antibiotics is an increasing problem in human health care^1^. By the year 2050, an estimated 10 million deaths worldwide per year could be attributed to bacterial infections^2^. The World Health Organization has declared multi-drug resistant (MDR) *Pseudomonas aeruginosa* a priority 1 critical pathogen^3^. Underpinning such concern is the paucity of new therapeutic agents in the development pipeline to treat infections with MDR *P*. *aeruginosa*. This is due, at least in part, to the highly impermeable outer membrane (OM) and robust efflux systems in this Gram-negative pathogen that together can prevent antibacterial small molecules from accumulating in cells sufficiently to engage intracellular targets. The OM is asymmetrical and consists of an inner leaflet of phospholipid and an outer leaflet of lipopolysaccharide (LPS). Biosynthesis of LPS and its assembly into the outer leaflet of the OM are essential for growth in most Gram-negative bacteria and have been the focus of intense interest for novel antibacterial discovery (e.g. the lipid A biosynthetic target LpxC^4^ and the OM assembly target LptD^5^).

LPS consists of a lipid A anchor, which forms the outer leaflet of the OM, attached to a polysaccharide core region linked to repeating polysaccharide O-antigen units that extend out from the cell surface. *P*. *aeruginosa* LPS is notable for its highly phosphorylated LPS core region^6^ and magnesium ions are thought to crosslink these phosphates to more tightly pack adjacent LPS molecules than in other Gram-negative bacteria. This serves to increase the OM permeability barrier which contributes to the high intrinsic drug resistance of *P*. *aeruginosa*^7^. Most of the enzymes catalyzing the biosynthesis of the core sugar moiety of LPS are encoded in a single gene cluster and may be associated in a protein complex^8^. This cluster includes at least three kinase genes (*waaP*, *wapP*, and *wapQ*) involved in phosphorylation of LPS core^7^. WaaP, a cytoplasmic soluble protein, phosphorylates the first heptose sugar of LPS inner core^9^, which appears to be required for subsequent core phosphorylation steps^10^. Lipid A core biosynthesis occurs in the cytosol with later steps, such as core phosphorylation (by WaaP and other LPS kinases), occurring on the lipid-A core associated with the inner leaflet of the inner membrane. This nascent LPS species is then flipped to the outer leaflet of the inner membrane where O-antigen is attached in the periplasm^11^. The fully decorated LPS molecule is then transported to the outer leaflet of the outer membrane through the Lpt transportation machinery^12^. Importantly, WaaP (and WapP) is essential for *P*. *aeruginosa* growth^7,10^ and as such constitute attractive anti-pseudomonal drug targets. The WaaP primary amino acid sequence shares homology to eukaryotic protein tyrosine kinases within its kinase active site^9^. The expanding knowledge of structure-based design of selective kinase inhibitors^13^ could be leveraged in the design of WaaP inhibitors. Here, we report the first X-ray crystallographic structure of *P*. *aeruginosa* WaaP, expressed in *Escherichia coli*, which revealed a very tight and novel complexation with *E*. *coli* acylated acyl-carrier protein (acyl-ACP). We further confirmed that acyl-ACP was indeed complexed with WaaP at physiologically relevant levels of WaaP expression in *P*. *aeruginosa*. ACP normally shuttles acyl chains (attached to ACP via a phosphopentatheine linkage) to various acyl-transferases involved in fatty acid^14^ and polyketide biosynthesis^15^, and the interaction of acyl-ACP with acyl-transferases is typically transient. However, here we discovered that acyl-ACP acts as a very tightly bound cofactor necessary for the production and stability of active WaaP kinase, uncovering a new function for acyl-ACP.

## Results

### Acyl-ACP/ WaaP crystal structure

We expressed C-terminal hexa-histidine tagged *P*. *aeruginosa* WaaP (1–264) in *E*. *coli* and purified it over four columns to near homogeneity (Supplementary Fig. 1). The purified WaaP kinase was active and transferred the γ-phosphate of ATP to LPS prepared from an *E*. *coli waaP* deletion strain, but not to fully-phosphorylated LPS prepared from *E*. *coli* wild type cells (Supplementary Fig. 1). Crystals of selenomethionine substituted protein were grown and data collected to 2.5 Å resolution (Supplementary Table 1). The anomalous diffraction signal from selenium identified a total of 6 sites in the asymmetric unit; however WaaP-His contained only 5 methionine residues. Initial solvent flattened electron density maps enabled the building of over 80% of the WaaP kinase structure but a large amount of unaccounted for density, containing the extra selenomethionine and a three-helical bundle, was evident. This unaccounted density was identified as being acyl carrier protein with an acyl chain modification (acyl-ACP). Purified WaaP-His run on gels designed to visualize low molecular weight proteins indeed showed a lower migrating band. Mass spectrometry (MS) analyses identified peaks with masses consistent with ACP protein carrying a C16:0 or C18:1 acyl chain from *E*. *coli*, confirming that acyl-ACP had been co-purified and co-crystalized with WaaP (Supplementary Fig. 1). The final crystal structure contained a 1:1 complex of *P*. *aeruginosa* WaaP and *E*. *coli* acyl-ACP.

**Figure 1:**
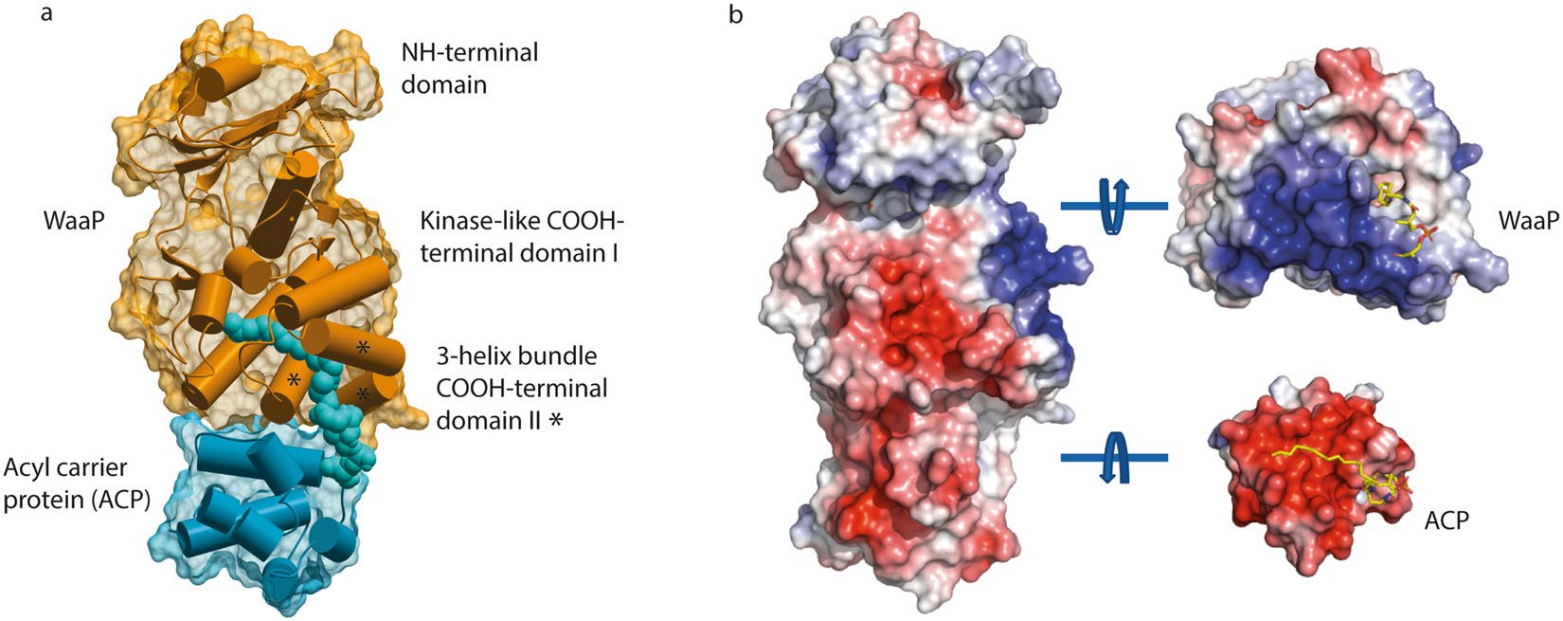
Crystal structure of the *P*. *aeruginosa* WaaP in complex with *E*. *coli* acyl-ACP. (**a**) A ribbon diagram and envelope representation of the WaaP-acyl-ACP complex show WaaP (orange) is comprised of an NH-terminal domain and COOH-terminal subdomains I and II (subdomain II is asterisked). The palmitoyl chain (space-filling model in cyan) attached to ACP (cyan) nestles through a hydrophobic tunnel encompassing both WaaP COOH-terminal domains and extends to the base of the adenine binding pocket. (**b**) Surface-potential map of the WaaP-acyl-ACP complex and rotation of the WaaP and ACP (right image) reveals protein-protein interface with extensive electrostatic interactions that occur over 645 Å with a basic (blue) WaaP surface and acidic (red) ACP surface. A stick model (yellow) attached to ACP is phosphopantetheine and a straight palmitoyl group.

The structural topology of the WaaP kinase consisted of a 6-stranded β-sheet NH-terminal lobe (1-118) flanked by two α-helices (19-24 and 73-89) with strong structural homology to eukaryotic protein kinase (EPK) family members, as expected from primary sequence analysis^16^, and a predominantly helical COOH-terminal lobe (119-263; Fig. 1). The COOH-terminal lobe was further divided into two sub-domains: the first sub-domain (I) (119-218) had structural homology to EPKs and the second sub-domain (II) (218-263) was composed of a novel arrangement of three helices, forming a channel through which a tube of electron density (approximately 20 Å long and 7 Å wide) extended into the COOH-terminal sub-domain I, ending under the kinase active site pocket. The density was consistent with a palmitoyl group (C16:0) on ACP. Weaker density matching a phosphopentatheine group extended from S36 in *E*. *coli* ACP that positioned the lipid palmitoyl group in the hydrophobic channel of *P*. *aeruginosa* WaaP. The WaaP COOH-terminal sub-domain II also had a large positively charged surface (approximately 800 Å^2^) defined by 12 surface exposed basic amino-acids: R221, R222, K224, R226, R229, R233, R234, R237, R241, R252, R253, and K256. A sub-set of these residues comprised the protein-protein interaction (PPI) surface engaging the negatively charged surface of ACP (see below).

ACP is a small (77 amino acid), highly negatively-charged (29% acidic residues) and ubiquitous (e.g ∼6 × 10^4^ molecules per *E*. *coli* cell) protein^14^ that delivers acyl-chains to enzymes of diverse function including those involved in polyketide^15^ and membrane biogenesis^17^. The ACP molecule bound to WaaP adopted a tertiary structure most similar to the form A crystals of butyryl-ACP (PDBID: 1l0h)^18^, with an RMSD of 0.7 Å^2^ for all Cα atoms, that reflected a closed and empty hydrophobic cavity within ACP. WaaP from *E*. *coli* and *P*. *aeruginosa* have previously been purified using *E*. *coli* overexpression systems and characterized biochemically, however the presence of ACP was not reported^9,19^. This is likely because it is a small (~9 kDa), acidic, and highly soluble protein that is difficult to visualize in typical SDS-PAGE analysis. WaaP was similarly not observed in MS-based proteomics of an ACP pulldown aimed at identifying ACP binding partners in *E*. *coli*^20^, perhaps due to limited sensitivity of the MS-based proteomics used in the experiments and possible low abundance of WaaP. We used the novel crystal structure identified here to guide the characterization of the kinase function of WaaP and to better understand the nature of its interaction with acyl-ACP.

### WaaP kinase domain

**Figure 2:**
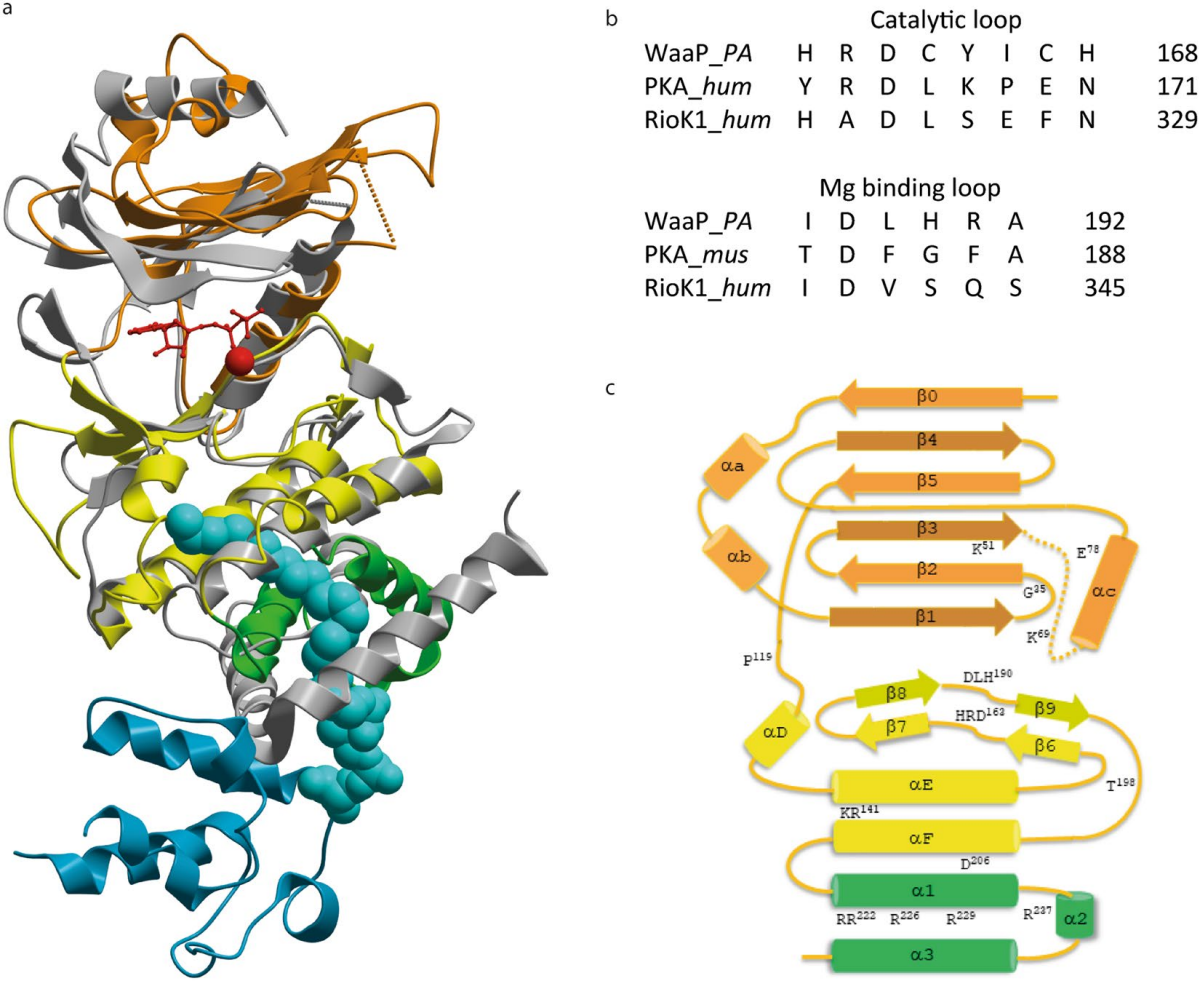
WaaP kinase domain shares significant structural homology with eukaryotic like protein kinases. (**a**) Superposition of crystal structure of WaaP/acyl-ACP and the kinase with most structural homology, the atypical kinase RioK1 (grey). WaaP is colored orange with the COOH-terminal sub-domain I is colored yellow and sub-domain II colored green. ACP is colored cyan and the palmitoyl chain depicted as a space-filling model. ADP (stick model) and Mg (space-filling model) bound to RioK1 are colored red. (**b**) Sequence alignment of kinase catalytic motifs for WaaP, PKA, and RioK1. Numbering from PDB entries 1ATP (PKA – mus musculus) and 4OTP (RioK1 - human). (**c**) Topology schematic of WaaP kinase. NH-terminal is colored orange, COOH-terminal sub-domain I colored yellow and sub-domain II colored green.

**Figure 3:**
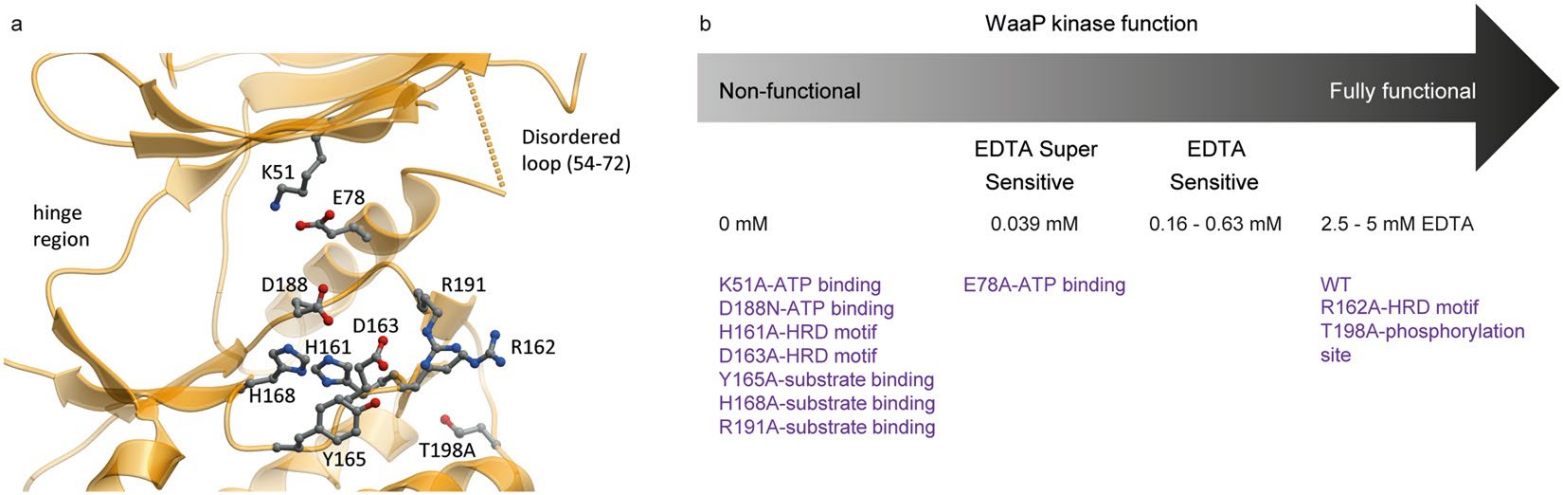
WaaP kinase active site. (**a**) A ribbon diagram of the WaaP kinase domain with a ball and stick representation of predicted key residues for WaaP kinase catalysis based on the structural homology to EPKs. (**b**) A graphical representation and categorization of EDTA MIC from a cell-based assay where WaaP variants were expressed and simultaneously WT WaaP was repressed in *P*. *aeruginosa* CDR0031. Variants were categorized by their viability in different concentrations of EDTA; no growth in LB (non-functional), in LB containing 0.039 mM EDTA (EDTA super sensitive), 0.16–0.63 mM EDTA (EDTA sensitive), or 2.5–5 mM EDTA (functional). EDTA sensitivity is indicative of kinase function. WaaP variants are annotated with their predicted function based on structural homology to EPKs. Data of WaaP functional assay using the cell-based assays in both *P*. *aeruginosa* and *E*. *coli* are shown in Table 1 and Supplementary Table 2.

We first sought to define residues necessary for WaaP kinase function based on structural homology to classic EPKs^21^. Previous reports have described WaaP as a novel dual function protein that can auto-phosphorylate at tyrosine residues and phosphorylate sugar (heptose I of LPS)^15^. We found no evidence of protein phosphorylation by MS analysis (for either WaaP or ACP) (Supplementary Fig. 1). Structurally, the EPK-family residues required for ATP binding and phosphotransferase were mostly present, however important differences were observed that placed WaaP in the eukaryotic protein kinase-like (ELK) family. Conserved residues included: the T114 gatekeeper residue; the hinge extending from E116 to P119, the “glycine-rich loop” contained a single glycine, G35, at its apex; the catalytically invariant lysine K51 was engaged by the side-chain of E78 from the NH-terminal of αC-helix. Residues D188, L189, and H190 represented the DFG motif on the Mg^2+^ binding activation loop^22^; and H161, R162 and D163 represented the HRD motif (Figs. 2, 3). These key kinase residues (K51, E78, H^161^RD, D^188^FG) were well conserved in WaaP homologs (Supplementary Fig. 2). The positioning of the catalytic lysine K51 (previously identified to be K69, see below) and D188 is consistent with an EPK-like mechanism of ATP α- and β-phosphate engagement and substrate hydroxyl interaction, respectively. Differences from classic EPK structure elements included the novel COOH-terminal sub-domain, a modified Mg^2+^ binding activation loop where histidine (H168 in WaaP) coordinated to the second Mg^2+^ ion in place of a conserved asparagine residue (N171 in protein kinase A), and a shortened, structured WaaP activation loop lacking EPK regulatory elements. This was consistent with a fact that we did not observe phosphorylation of WaaP. The residues in the activation loop normally modified by phosphorylation to enable regulation of catalytic activity were replaced by two buried threonine residues (T194 packed against αC-helix and T198). The key α-helical elements involved in catalytic activity from the C-terminal lobe of EPK family members (on helices αE- and αF) were present in WaaP, such as the D206 (from αF-helix) interaction with the main-chain of H161 in the HRD motif (Fig. 2). However, the GHI-helical subdomain responsible for the regulation and substrate recognition of EPK proteins^23^ was replaced in WaaP by a novel 3-helical fold comprising the acyl-ACP interacting COOH-terminal sub-domain II of WaaP. The acyl chain attached to ACP projected between αE- and αF helices (see below). No residues were observed that would indicate an acyl transfer capacity for WaaP. The observations derived from this novel co-structure showed that WaaP was a member of the ELK family of proteins^24^ as exemplified by the structural similarity to RioK1, an atypical kinase^25^ (Fig. 2).

To directly assess our structure-based predictions on the function of kinase domain residues, we interrogated the impact of alterations of a subset of these residues on WaaP function in a cellular context. To accomplish this, WaaP variants were expressed in a previously described *P*. *aeruginosa waaP* controlled expression strain (CDR0031)^10^. Since WaaP is essential for growth in *P*. *aeruginosa*, the ability to turn off expression of the native WaaP provided a way to assess whether WaaP variants were functional, based on their ability to support growth. Downregulation of native WaaP expression also increased the cells susceptibility to EDTA^10^, where presumably a lower EDTA MIC correlates to a lower amount of phosphorylated LPS in the outer membrane. This enabled more subtle defects in the activity of WaaP variants to be detected via increased susceptibility to EDTA. The impact of amino acid substitutions at several key residues (K51A, E78A, H161A, R162A, D163A and D188N) on bacterial growth and susceptibility to EDTA compared to wild type (WT) WaaP is shown in Fig. 3. As expected, cells expressing WT WaaP grew and were relatively resistant to EDTA (minimal inhibitory concentration (MIC) of 5 mM). Most of the variants of structurally-predicted essential kinase residues could not support growth and cells expressing the E78A WaaP variant were at least two orders of magnitude more sensitive to EDTA (MIC < 0.04 mM EDTA; Fig. 3). However, expression of the R162A variant did not alter susceptibility to EDTA (Table 1 and Fig. 3) and the T198A variant behaved like WT WaaP, consistent with the absence of phosphorylation-mediated kinase regulation^26^. Western blot analysis showed that all of these variants were produced in cells at levels comparable to the WT WaaP (Supplementary Fig. 3), indicating that protein stability was unaffected. Residue K69 was assigned previously as the catalytic lysine based on primary sequence alignments and mutational analysis^9^. This residue was part of a disordered loop (54–72, Fig. 3a) at the NH-terminal of the αC-helix of WaaP. Protein variants in this loop have previously been reported to impact function in multiple protein kinase oncogenes, including *ERBB2*^27^. The K69A variant had a modest (4-fold) increased EDTA sensitivity, in line with previous reports^9^ (Table 1 and Supplementary Table 2). As predicted by the structural analysis, WaaP altered at the catalytic K51 residue (K51A) did not support growth of *P*. *aeruginosa*, confirming that K51 is critical for WaaP function. To further support these findings, we exploited the observation that, although WaaP is not essential for growth of *E*. *coli*^28^, cells lacking WaaP are more sensitive to novobiocin (NOV)^9^. Since wild type *P*. *aeruginosa* WaaP can functionally complement *E*. *coli* WaaP^19^, we could also determine if the WaaP variants were active in an *E*. *coli waaP* deletion strain. As expected, expression of wild type *P*. *aeruginosa* WaaP in the *E*. *coli waaP* cell background restored NOV resistance whereas the variants altered at key kinase domain residues did not (Supplementary Table 2). Therefore, the results of both *P*. *aeruginosa* and *E*. *coli* cellular assays confirmed that WaaP shared EPK kinase mechanisms that are critical for ATP phosphotransferase function but does not possess the regulatory mechanisms typical of EPK family members placing WaaP in the ELK family. Furthermore, the results show that the kinase function of WaaP is essential for *P*. *aeruginosa* growth and EDTA resistance, and proper maintenance of the *E*. *coli* OM permeability barrier, as reflected by NOV resistance.

**Table 1:** Cell-based assay for WaaP variants expressed in WaaP controlled expression strain in *P*. *aeruginosa* assessed for minimal inhibitory concentration (MIC) of EDTA.

WaaP variant	Rationale	*P*. *aeruginosa* EDTA MIC fold shift^*^
WT	Wild type	0.5
Δ*waaP* or pAK1900	WaaP depletion or vector control	no growth
pAK1900-WaaP-WT	complementation	1
K51A	ATP binding	no growth
K69A	ATP binding	4
E78A	ATP binding	128
D188N	ATP binding	no growth
T198A	phosphorylation site	1
H161A	HRD motif	no growth
R162A	HRD motif	2
D163A	HRD motif	no growth
Y165A	substrate binding	no growth
H168A	substrate binding	no growth
R191A	substrate binding	no growth
R222A	ACP binding	8
R222E	ACP binding	2
R226A	ACP binding	1
R226E	ACP binding	1
R222/226A	ACP binding	2
R222/226E	ACP binding	16
R222/226/237E	ACP binding	128
R221/222/226/229/237E	ACP binding	no growth
L143W	lipid terminus	2
S127L	lipid terminus	2
L228A	pocket proximal to phosphopentatheine	2
L228W	pocket proximal to phosphopentatheine	1
L219W	mid pocket	1
V147L	mid pocket	8
V147W	mid pocket	no growth
A214L	mid pocket	16
A214F	mid pocket	2
A214W	mid pocket	16
V147L/L219W	mid pocket	8
V147W/214F	mid pocket	no growth

^*^MIC was the mode of values (a minimum of biological triplicate) defined by the minimum inhibitory concentration of chemical to inhibit 90% of growth compared to cells grown in LB in the absence of chemicals. The raw MIC values are shown in Supplementary Table 2.

### Role of acyl-ACP/WaaP interaction

**Figure 4:**
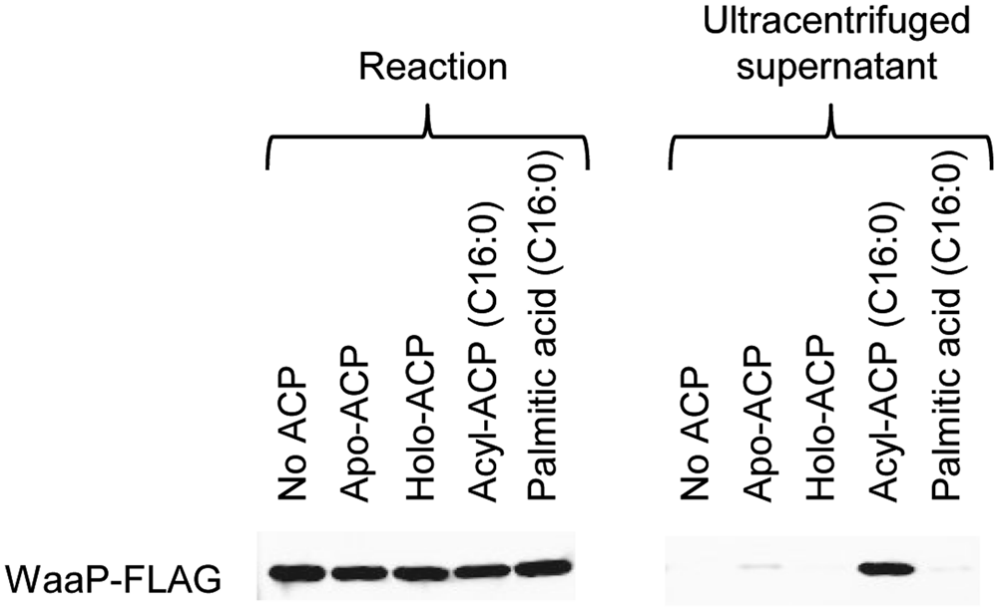
*In vitro* synthesis of soluble WaaP in the presence of acyl-ACP. WaaP-FLAG was expressed from a DNA fragment containing P_T7_::*waaP*-FLAG using an *in vitro* transcription and translation system in the absence of *E*. *coli* ACP or in the presence of apo-ACP (unmodified ACP), holo-ACP (phosphopentheinylated ACP), palmitoyl-ACP, or free palmitic acid. Immunoblotting with anti-FLAG antibody was used to visualize expressed WaaP in the reaction samples (left) and in the supernatant of the ultracentrifuged sample (right). The uncropped gel of the immunoblot and the SDS-PAGE gel of proteins stained with SyproOrange are shown in Supplementary Fig. 4.

The WaaP/acyl-ACP crystal structure showed an extensive PPI surface combined with insertion of the acyl-ACP lipid chain into the WaaP hydrophobic channel which implied an unusually strong interaction compared to other characterized ACP complexes. We hypothesized that acyl-ACP was therefore necessary for the production and maintenance of stable and soluble WaaP. To test this, we employed an *in vitro* transcription/translation system to produce WaaP-FLAG with and without the presence of purified enzymatically acylated *E*. *coli* ACP. A similar level of WaaP-FLAG protein was produced regardless of the presence or absence of only detected in the supernatant after ultracentrifugation when acyl-ACP was present, as shown by anti-FLAG western blot (Fig. 4) and SYPRO Orange stained protein gel (Supplementary Fig. 4). WaaP produced under all other conditions was pelleted by utracentrifugation suggesting that, in the absence of acyl-ACP, WaaP was either associated with larger cellular components or aggregated. This supports the hypothesis that acyl-ACP is required for the production of soluble, stable WaaP.

### Analysis of Acyl-ACP/WaaP interaction

**Figure 5:**
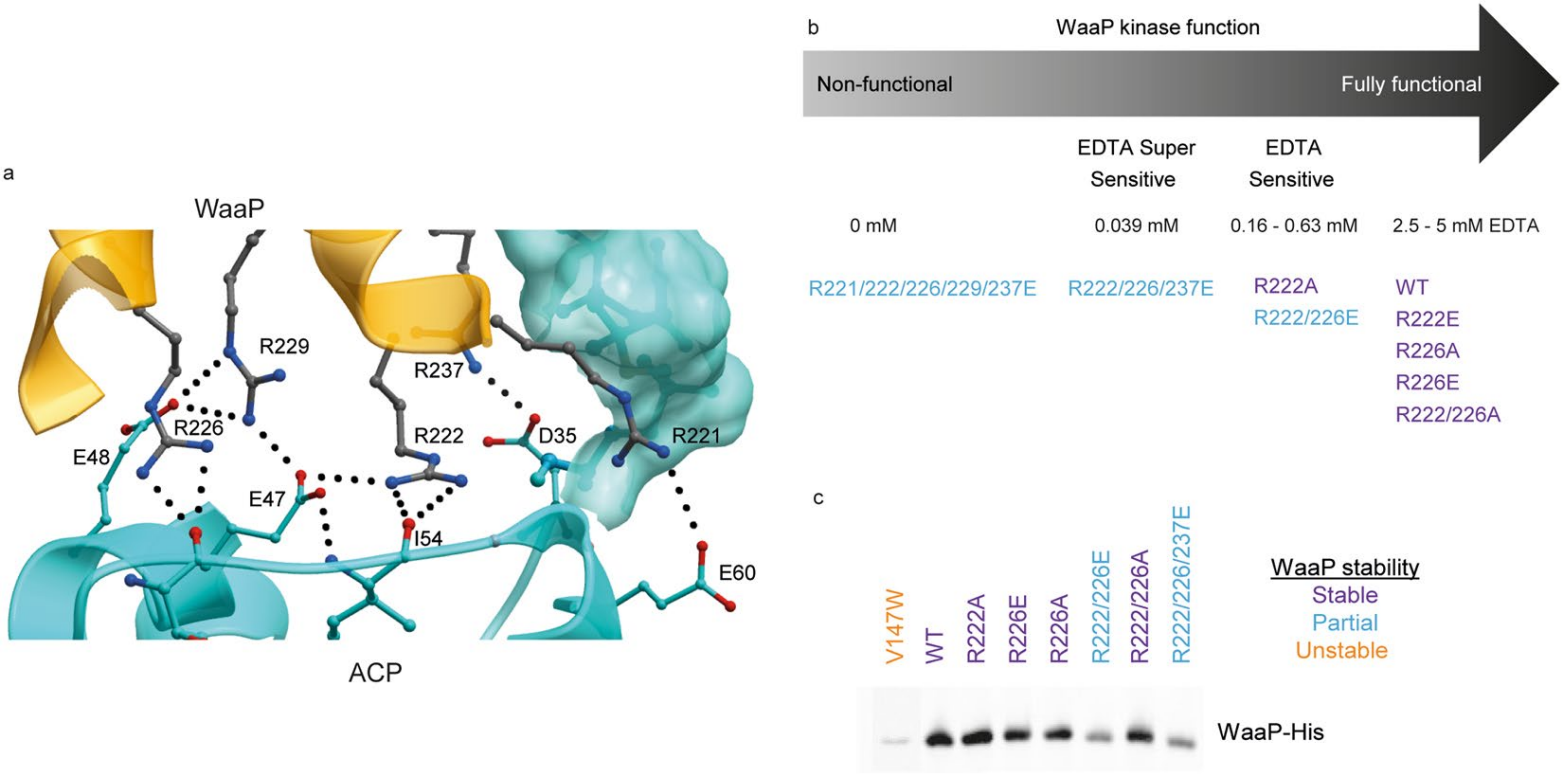
Protein-protein interaction interface between acyl-ACP and WaaP. (**a**) A ribbon diagram of the WaaP COOH-terminal domain II (orange) and acyl-ACP (cyan). Residues involved in the interaction are shown with ball and stick visualization and an acylated phosphopantetheine attached to ACP is shown as a Van der Waals surface. (**b**) WaaP kinase function comparing WaaP wild type (WT) and variants with amino acid substitutions from Arg to either Ala or Glu in WaaP’s protein interaction surface in the cell-based assay described in Fig. 2b. (**c**) Anti-His immunoblot shows relative stability of WaaP-His variants in *P*. *aeruginosa* cells. Protein stability is classified as stable (purple), partially stable (blue), unstable (orange). The color classification is applied to Figs. 2b, 4b, and 5b. The anti-His immunoblots of all WaaP variants are shown in Supplementary Fig. 3.

**Figure 6:**
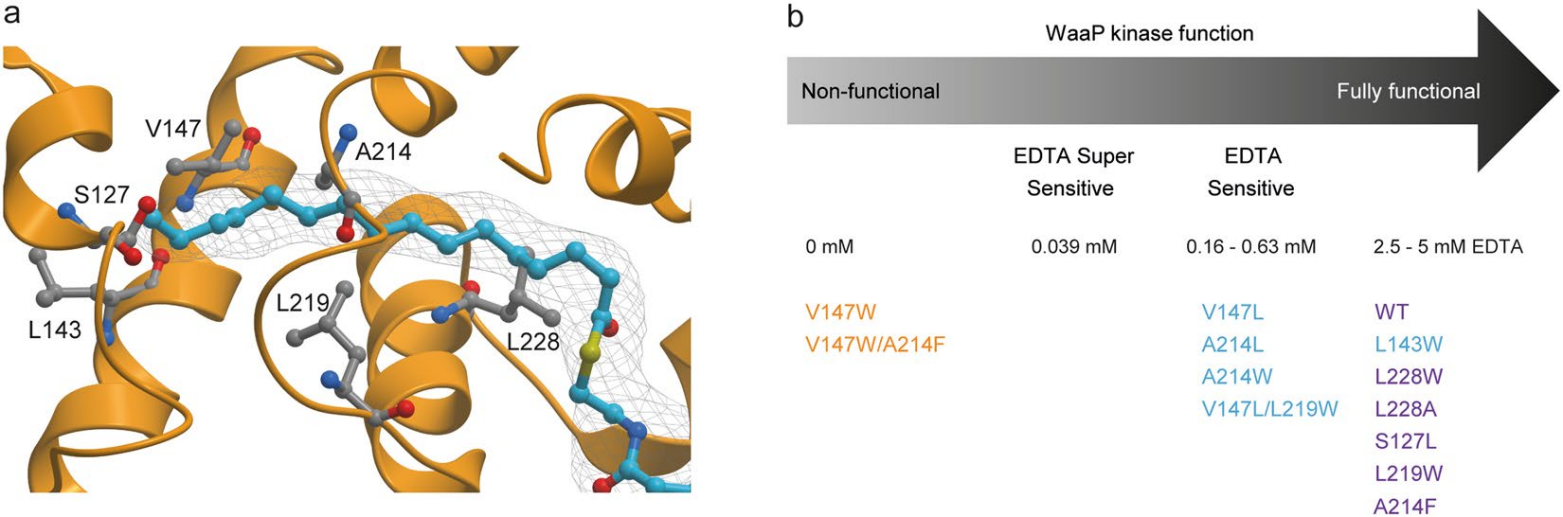
Interaction between the acyl chain on ACP and the hydrophobic tunnel of WaaP. (**a**) A ribbon diagram of WaaP (orange) and the acyl chain moiety (cyan) of acyl-ACP. A SAD-phased, density modified map contoured at 1.4σ is shown as a mesh around the acyl chain. (**b**) WaaP kinase function determined by EDTA MIC and stability comparing WaaP wild type (WT) and variants with amino acid substitutions of residues in the hydrophobic tunnel of WaaP in the *P*. *aeruginosa* cell-based assay. Protein stability is shown as stable (purple), partially stable (blue), unstable (orange).

The WaaP/acyl-ACP interface was composed of two discreet interactions: a ~645 Å^2^ hydrophilic PPI and the insertion of the lipid chain of acyl-ACP into a ~680 Å^2^ hydrophobic invagination in the COOH-terminal lobe of WaaP (Figs. 1, 5, and 6). The hydrophilic PPI was formed by interactions between the negatively charged surface of ACP (residues D35, E41, E47, E48, E53 and D56) with a positively charged surface on WaaP (residues K140, R141, R221, R222, R226, R229 and R237). The acyl-ACP/WaaP interaction could not be characterized by the simple deletion or depletion of ACP, since ACP interactions with a range of protein partners are necessary for cell growth^17^. Therefore, we chose to make variants of WaaP to evaluate the importance of the positively charged PPI surface for WaaP function and stability in our cell-based assays. As described above, kinase function was measured as the ability of WaaP variants to support growth of *P*. *aeruginosa* and to mediate EDTA resistance, and stability was estimated by western blot analysis. WaaP variants designed to disrupt the interface, by substituting neutral Ala or charge-inverted Glu residues, were generated both individually and in combination and assessed (Fig. 5; Supplementary Table 2). The WaaP variants R222E, R226E, and R222A/R226A were stable and supported cell growth and wild type levels of EDTA susceptibility. The double R222E/R226E variant was less stable in cells (but still supported growth), however, the susceptibility of cells to EDTA was increased 16-fold consistent with a reduced kinase activity (Fig. 5). Enriching for substitutions from positively charged Arg to negatively charged Glu within the PPI (R222E/ R226E/R237E and R221E/R222E/R226E/R229E/R237E) progressively decreased WaaP stability and cellular function to the point that R221E/R222E/R226E/R229E/R237E could not support growth in the WaaP controlled expression strain. This indicated that the WaaP protein-protein interaction with ACP is necessary to maintain active WaaP and furthermore, as predicted, the interaction is very difficult to disrupt. As with the analyses of the kinase domain above, the results were mirrored by the orthogonal *E*. *coli* cellular assay (Supplementary Table 2), further supporting the notion that disrupting interaction at the WaaP/ACP PPI decreased soluble and functional WaaP protein levels leading to a corresponding reduction of membrane integrity.

We next examined residues within the hydrophobic channel of WaaP where the palmitoyl acyl chain of ACP was bound in the crystal structure. WaaP variants altered at residues in the hydrophobic channel were designed to perturb the regions corresponding to the terminal end of the lipid (L143W, S127L), the mid-pocket area (L219W, V147L, A214L, A214W, V147W, V147L/L219W, V147W/A214F) and the solvent-exposed opening of the tunnel proximal to the phosphopantetheine-ACP acyl chain attachment site (L228A, L228W). Changes at either end of the hydrophobic tunnel had little impact on WaaP stability and function (S127L, L228A, L228W). Variants altered at residues lining the middle of the hydrophobic tunnel ranged from fully stable and functional to non-stable and non-functional (e.g. V147W and V147W/A214F, predicted to bisect the hydrophobic tunnel) (Fig. 6). In general, the ability of WaaP variants to support cell growth correlated with their relative stability as measured by western blot. As above, largely similar results were obtained using the *E*. *coli* system, although WaaP variants expressed in *E*. *coli* appeared overall to be even less tolerant to changes in mid-pocket residues (Supplementary Table 2). Intriguingly, dramatic hydrophobic core substitutions such as L219W were tolerated, suggesting that WaaP stability/function does not depend on a specific acyl-chain length. While not definitive, this suggests that the acyl-chain interaction can be promiscuous but is required for WaaP protein folding and stability.

### Myristyl-AcpP/WaaP complex in *P. aeruginosa*

**Figure 7:**
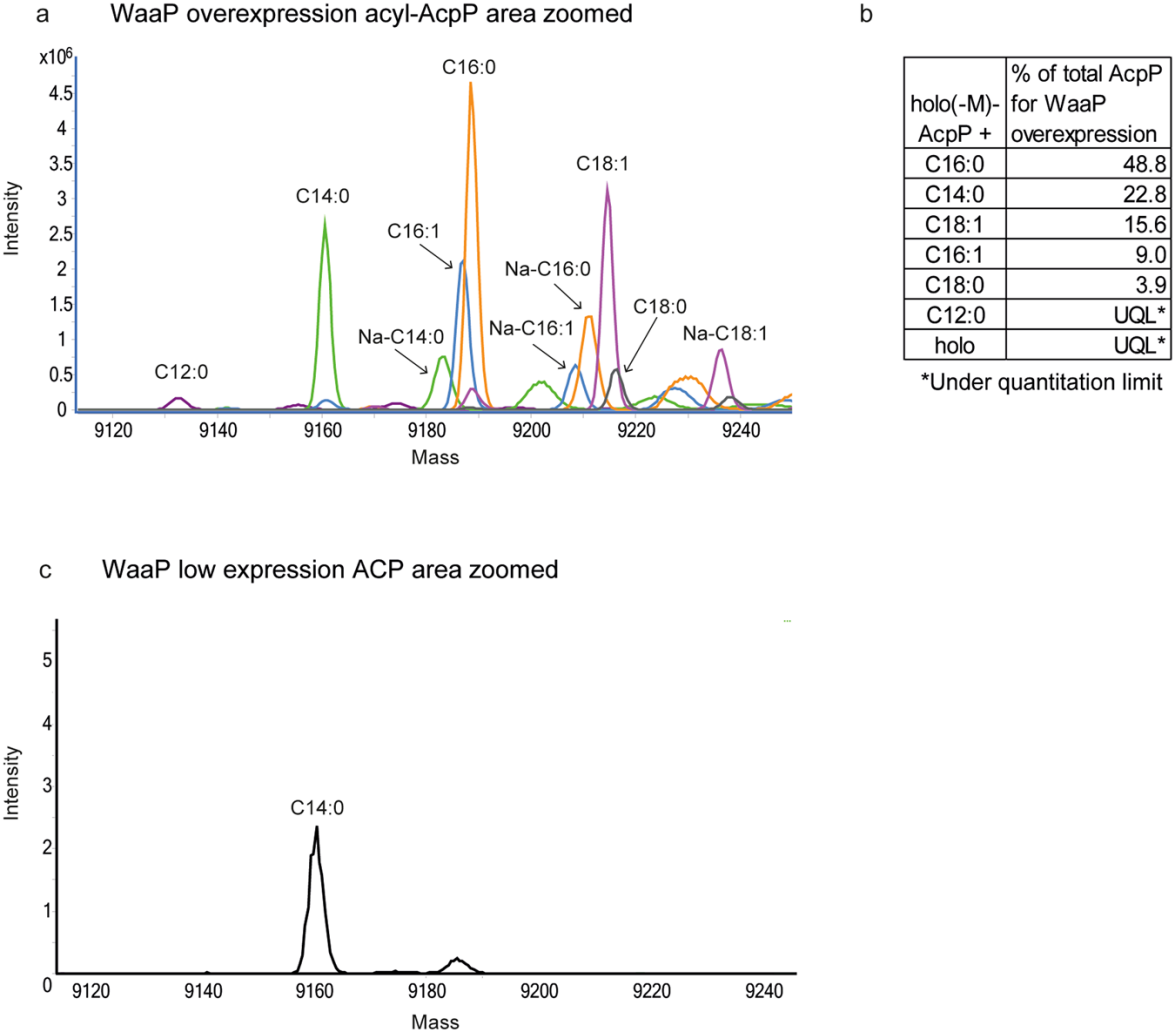
Mass spectrometry identification of acyl-AcpP in samples pulled down by WaaP-His from *P*. *aeruginosa*. (**a**) Overlaid LC-MS protein spectrum (shown in different colors) at the mass range of acyl-ACP species in samples pulled down by WaaP-His from *P*. *aeruginosa*. Mass peaks consistent with acyl-AcpP (-Met) are labeled with the acyl chain moiety of acyl-AcpP. The detailed peak assignments are shown in Supplementary Table 4. (**b**) Ratio of the amounts of AcpP species detected in Fig. 6a. The peak area for each individual acyl-AcpP species (total of salt-free acyl-AcpP and that with sodium adducts) was divided by the total peak area for all acyl-AcpP species to determine the percentage of each acyl-AcpP. (**c**) LC-MS protein spectrum at the mass range of acyl-ACP species in the WaaP-His pull-down samples from *P*. *aeruginosa* cells expressing WaaP-His 40-fold lower than the sample shown in Fig. 6a shows peak consistent with only C14-AcpP. The small peak at 9195 was not consistent with any AcpP species. The MS spectrum for WaaP-His in the pull down samples are shown in Supplementary Fig. 5a,b.

*E*. *coli* has only one ACP, and as such *P*. *aeruginosa* WaaP overexpressed in *E*. *coli* was bound to that specific ACP. In contrast, *P*. *aeruginosa* contains 3 ACP paralogues: AcpP, Acp1, and Acp3. AcpP is the closest homolog of *E*. *coli* ACP with 88% sequence identity and is essential for growth of *P*. *aeruginosa*, whereas Acp1 and Acp3 are more divergent and not essential for growth^29^. Structural alignments of the ACP proteins with *E*. *coli* ACP (not shown) suggested that AcpP and Acp1, but not Acp3, possessed residues that could potentially interact with WaaP. To confirm the existence of an acyl-ACP/WaaP complex and identify the specific acyl-ACP species bound to WaaP in the physiologically relevant context of *P*. *aeruginosa* cells, His-tagged WaaP was expressed and pulled down from exponentially growing *P*. *aeruginosa* cells using IMAC followed by MS analysis of co-eluted proteins. MS peaks consistent with WaaP (31,572 Da) (Supplementary Fig. 5a) and additional peaks were observed with masses clustering around 9200 (Fig. 7a). The protein masses of the peaks matched acyl-AcpP carrying several acyl chain lengths whereas masses consistent with Acp1 and Acp3 were not observed. Overnight treatment of the sample with DTT to cleave the thioester bond and release the acyl chain caused the collapse of MS peaks assigned as acyl-AcpP species into a single peak for holo-AcpP, confirming the MS peak identification of the various acyl-ACP species (Supplementary Fig. 5c,d). This suggested that WaaP can bind various acyl-AcpP species, consistent with our observation above that the WaaP hydrophobic channel could tolerate amino acid substitutions. The most abundant ACP species pulled down from *P*. *aeruginosa* was palmitoyl (C16:0)-AcpP (Fig. 7). Palmitic acid is the most abundant fatty acid in the cell^30^ and this chain length is consistent with the acyl chain of acyl-ACP assigned in the crystal structure. Intriguingly, the second most abundant species was myristyl-AcpP (C14:0; Fig. 7), even though myristic acid comprises only 0.6% of the fatty acid pool in *P*. *aeruginosa* cells^30^. Furthermore, WaaP-His expressed at ~40-fold lower protein levels (Supplementary Fig. 5) co-eluted only with the myristyl-AcpP as measured by MS (Fig. 7). This suggests that WaaP acquired abundant acyl-ACP species when overexpressed but preferentially bound myristyl-ACP at lower expression levels which may be more reflective of endogenous WaaP levels. Since the peak was small, we confirmed the presence of AcpP by trypsin digest and MS/MS peptide analysis (Supplementary Table 3). There was no evidence for phosphorylation of AcpP or acyl transfer to WaaP, consistent with our hypothesis that acyl-AcpP stabilizes WaaP rather than acting as a substrate. Our results show that WaaP interacts with acyl-AcpP in *P*. *aeruginosa* and the WaaP hydrophobic channel can accommodate acyl chains of different lengths, but appears to preferentially bind myristyl-ACP at physiologically relevant levels of WaaP.

## Discussion

Here we report a novel and unexpected complex between the *P*. *aeruginosa* LPS core heptose kinase WaaP and acyl-AcpP that raises several intriguing implications. The bacterial kinome is composed of protein kinases and sugar kinases with ancestral sequences and structural homologies to eukaryotic kinase families^16,23^. The WaaP crystal structure showed a protein fold that is homologous to EPKs in the NH-terminal lobe and sub-domain I of the COOH-terminal lobe. The catalytic core of the protein kinase family was mostly conserved and demonstrated to be essential for WaaP cellular function, suggesting a conserved EPK-like mechanism of ATP dependent phosphotransfer. The canonical EPKs also contain regulatory mechanisms mediated by the catalytic loop which were not present in WaaP. The human kinome has been annotated to contain several protein kinase families that similarly diverge and WaaP shared the most structural similarity with the Rio family of atypical kinases^25^ (Fig. 2). WaaP also diverged from known kinase structures in the motifs in the second-half of the COOH-terminal lobe known to be responsible for kinase substrate recognition. A unique arrangement of 3 helices replaced the canonical EPK GHI-helical subdomain^31^. This unique sub-domain II of WaaP’s COOH-terminal lobe formed an internal channel, where the acyl chain attached to ACP was buried. WaaP presented a highly positively charged polar protein surface where ACP made a highly charged-complimentary PPI. The remaining solvent exposed positively-charged surface of WaaP is presumably required for recognition of the negatively-charged substrate (lipid A-core-sugar) similar to the site postulated for substrate recognition of the LPS core sugar glycosyltransferase, WaaG^32^.

We initially uncovered the WaaP/acyl-ACP interaction while solving the WaaP crystal structure utilizing an *E*. *coli* overexpression system. We then confirmed that it was physiologically relevant in *P*. *aeruginosa* cells using a variety of approaches such as *in vitro* protein synthesis, pull-down experiments, and mutational analyses. WaaP altered at canonical kinase catalytic resides could be expressed stably in *P*. *aeruginosa* but were unable to support growth and *E*. *coli* expressing these variants were more susceptible to NOV, consistent with the importance of the kinase function in OM biosynthesis and permeability.

The interaction between WaaP and acyl-ACP was predicted from the structure to be much tighter than would be expected for typical interactions of acyl-ACP as a substrate, which must be transient to allow for catalytic turnover (e.g. acyltransferases). PPI regions of ACP-utilizing enzymes studied so far have at most 3-4 positively charged residues^33–38^, whereas the interaction of WaaP with acyl-ACP was mediated by 7 positively charged Arg and Lys residues, providing extensive hydrogen bonding over a large surface area. Further hydrophobic interactions between the acyl chain and residues in the hydrophobic tunnel of WaaP serve to strengthen the acyl-ACP/WaaP association. Consistent with this, the complex survived extensive purification under high salt conditions and pulldown experiments invariably yielded an acyl-AcpP/WaaP complex. The complex PPI region of WaaP had to be extensively altered to observe an impact on the stability of acyl-AcpP/WaaP and correspondingly its ability to support cell growth. Furthermore, soluble WaaP could only be synthesized *in vitro* in the presence of acyl-ACP. This unusually strong protein-protein interaction therefore is required to maintain WaaP in a soluble active state.

It is always tempting to propose a regulatory role in cases of protein-protein interactions. For example, acyl-ACP is a suspected regulator of SpoT based on protein associations^35^ and this may also be the case here. The connection between LPS synthesis (lipid A) and phospholipid synthesis, which both utilize acyl-ACP, and the need to balance these processes to maintain inner and outer membrane homeostasis^39,40^, may support such possibilities. Furthermore, *P*. *aeruginosa* LPS contains only C10:0 and C12:0 acyl chains whereas quantifiable acyl-ACP species associated with WaaP pulled down from *P*. *aeruginosa* contained longer acyl chains (C14:0 – C18:0), supporting the possibility that WaaP stability is linked to a pathway other than LPS biosynthesis. Any such interrelationships, and the possible role of factors controlling the abundance of various acyl-AcpP species that may control WaaP function would warrant further study. In addition, the interaction with acyl-ACP mediated by the unique kinase structural motifs of WaaP could conceivably regulate enzyme function and substrate recognition. Another possibility is that this interaction has evolved primarily to maintain a pool of active, but potentially low-abundance, WaaP via this extremely stable complex. The unusually large PPI and hydrophobic tunnel region mediating the interaction may suggest continual selective pressure favoring increased tightness over time. The interaction is clearly unrelated to acyl-transfer normally mediated by acyl-ACP and indeed the acyl chain is itself specifically exploited to strengthen the interaction with WaaP, with the acyl chain being protected in a dedicated hydrophobic tunnel. We postulate that synthesis and preservation of a pool of active WaaP is paramount in *P*. *aeruginosa* and that WaaP exploits a generally abundant protein partner^14^ to stabilize the low abundant WaaP in an active WaaP/acyl-ACP complex.

We show that both the kinase function and the interaction with acyl-AcpP with WaaP are necessary for growth of *P*. *aeruginosa*, and disruption of either of these could constitute a target for the development of novel therapeutics for treating infections. However the complexity and extreme tightness of the acyl-AcpP interaction uncovered here suggests that identifying small molecule inhibitors of that interaction may be challenging^41^. Conversely, the structural and sequence understanding of the ATP binding pocket of EPKs has been successfully exploited for oncology therapies^42^ and antibacterials^43^ combined with the apparent biological imperative to preserve active WaaP in cells, supports an opportunity to target the WaaP kinase domain for the development of novel anti-pseudomonal agents.

## Methods

### General methods

Full description of methods is provided in Supplementary Information. *P*. *aeruginosa* strains and *E*. *coli* strains were derived from the prototrophic strain PAO1 and the K-12 strains, respectively. Unless otherwise noted, cells were grown at 37 °C in Lysogeny broth (L broth or LB) or LB supplemented with the appropriate antibiotic for the plasmid and strain. Plasmids were constructed using PCR, synthetic DNA, ligation, and Gibson assembly. Point mutants were generated using NEBaseChanger for primer design and the Q5 site-directed mutagenesis kit. Transformation was done with electroporation. Electrocompetant *E*. *coli* strains were made by washing overnight cultures 5 times with ice cold 10% glycerol and *P*. *aeruginosa* strains were washed 3 times with 300 mM sucrose at room temperature^44^. Detailed cloning and strain construction description are provided in Supplementary Information. Lists of strains (Supplementary Table 5), plasmids (Supplementary Table 6) and oligonucleotide primers (Supplementary Table 7) can be found online as Supplementary Information. Protein purification was performed using FPLC (GE Life Sciences) or with gravity-flow.

### Expression and purification of WaaP for crystallography

*E*. *coli* BL21(DE3)/pLysS was freshly transformed with pET21b-*waaP* [*P*_*T7*_*::PawaaP-his6*]. The fresh transformants were inoculated in SelenoMet medium base (Molecular dimensions) supplemented with 100 µg/mL carbenicillin (Cb), 34 µg/mL chloramphenicol (Cm), and 8 µg/mL methionine. After overnight incubation, the cultures were centrifuged and the pellets were resuspended in fresh medium 100 µg/mL Cb, 34 µg/mL Cm, and 8 µg/mL selenomethionine. Cells were grown to the OD_600_ of 0.6 and cooled to 25 °C prior to addition of 1 mM IPTG and grown for 4 hours at 25 °C. The cells were harvested by centrifugation at 250 rpm and resuspended (5 mL per gram cell pellet) in buffer A (50 mM Tris-HCl pH 8.0, 500 mM NaCl, 50 mM arginine, 50 mM glutamic acid, 1 mM TCEP) supplemented with 20 mM imidazole, 1 mM PMSF, and complete Protease Inhibitor Cocktail Tablets (1 tablet/50 mL - Roche Biochemicals). The cell suspension was homogenized on ice using a Polytron Mechanical Homogenizer (1 × 30 sec) and then lysed with a micro-fluidizer (Microfluidics). The lysate was clarified by ultracentrifugation (138,000 × *g*, 60 min, 4 °C). WaaP-His was affinity purified with IMAC sepharose fast flow resin and further purified by ion exchange chromatography using Mono-Q and CM-sepharose columns followed by size-exclusion chromatography using a Superdex75 column.

### Crystallization and structure determination

WaaP-His in buffer B (20 mM Tris-HCl pH 8.0, 100 mM NaCl, 500 mM ammonium acetate, 1 mM TCEP) was mixed with equal volumes of crystallization buffer (100 mM HEPES-NaOH pH 7.4, 5% Jeffamine M-600) and crystals were grown via hanging drops at 4 °C over the course of 48 hours. Crystals were flash frozen using 20% ethylene glycol as a cryo-protectant and data collected at 100 K with monochromatic X-rays at a wavelength of 0.9791 Å using a Dectris Pilatus 6 M detector on the PXII-X10SA beamline at the Swiss Light Source, Paul Scherrer Institut, Villigen, Switzerland. Data were integrated and scaled using the *XDS* package^45^ and selenium sites identified using the SHELX software package^46^. Data diffracted to 2.5 Å, albeit with a high Wilson B-factor of 69.5 Å^2^, and a space group of P3_1_21 and unit cell dimensions of a = 92.023 Å, b = 92.023 Å, c = 99.172 Å. The electron density map generated using this information combined with solvent flattening restraints was of such quality that ~80% of the protein structure could be readily identified. Model building and refinement were carried out using COOT^47^ and PHENIX^48^. Final statistics are shown in Supplementary Table 1. Atomic coordinates and structure factors are deposited in the Protein Data Bank with the accession number 6DFL.

### Acylation of holo-ACP

Description of expression and purification of *E*. *coli* holo-ACP and *Vibrio harveyi* acyl-ACP synthetase is provided in Supplemental Information. The acylation of holo-ACP by AasS was performed with incubation for 2 hours at room temperature (completion was monitored by MS) in a final volume of 160 µL containing 50 mM MOPS-NaOH pH 7.5, 5 mM MgSO_4_, 5 mM ATP, 0.5 mM palmitic acid (100 mM stock in methanol), 1 mM TCEP, 0.16 mM holo-ACP, and 160 nM AasS. The buffer was exchanged to buffer (20 mM MOPS-NaOH pH 7.5, 500 mM NaCl, 1 mM TCEP) using a PD-10 desalting column. Palmitoyl-ACP was concentrated using an Amicon Ultra-4 centrifugal filter unit MWCO 3000.

### *In vitro* protein synthesis

PURExpress *in vitro* protein synthesis Kit (New England Biolabs) was used following the instruction with modifications. A PCR product containing a T7 promoter, codon optimized *waaP-*FLAG, and a T7 terminator. The reaction was incubated at 37 °C for 2.5 hours in a final volume of 100 µL containing 10 ng/uL DNA template, 0.8 units/uL murine RNase inhibitor, solution A and solution B, and either no ACP, or 7.6 ng/µL apo-ACP, holo-ACP, or palmitoyl-ACP, or 200 µM palmitic acid. To separate soluble WaaP-FLAG from protein aggregates including unstable WaaP, 85 µL reaction samples containing synthesized WaaP-FLAG were ultracentrifuged at 350,000 × *g* for 15 min at 10 °C in a TLA 100.2 rotor using Beckman Optima TLX Ultracentrifuge. Samples were analyzed using SDS-PAGE stained with Sypro Orange and Western blotting with mouse anti-FLAG primary antibody (DYKDDDDK tag antibody, Invitrogen, MA1-91878) and IRDye 800 CW donkey anti-mouse IgG (IRDye 800CW Donkey anti-mouse, Li-cor, 926-32212).

### Cell-based functional assay for WaaP variants in *P. aeruginosa*

The WaaP functional assays in *P*. *aeruginosa* were performed using growth and EDTA MIC of *P*. *aeruginosa waaP*-controlled expression strain (CDR0031)^10^. CDR0031 were transformed with plasmids expressing WaaP wild type and variants in biological triplicates. BBL Prompt inoculation system was used to make standardized cell suspensions from single colonies. 1000x diluted cell suspensions were added to LB supplemented with two-fold serial dilutions of EDTA in 96-well microtiter plates and incubated overnight at 37 °C. EDTA MIC was defined as the lowest concentration of EDTA at which less than 10% of the OD_600_ in the control well (full growth) was measured.

### Immunoblotting to monitor expression levels of WaaP variants

LB cultures supplemented with 0.2% arabinose and 100 µg/mL Cb were inoculated from overnight cultures of *P*. *aeruginosa* CDR0031 carrying pAK1900-*waaP* plasmids expressing wild type WaaP-His6 and variants and grown at 37 °C to mid-exponential phase (OD_600_ = 0.4 − 0.6). The cultures were pelleted and resuspended in Laemmli sample buffer diluted with 50 mM MOPS-NaOH pH 7.0 and the samples were boiled. Samples normalized by cell density (OD_600_) were analyzed using SDS-PAGE stained with Coomassie Brilliant Blue Gels and immunoblotting visualized with mouse monoclonal anti-His primary antibody (THE^TM^ His-tag Antibody, GenScript, A00186) and IRDye 800 CW donkey anti-mouse IgG.

### WaaP-His pulldown in *P. aeruginosa*

Cells were grown with 1% inoculum from overnight culture of *P*. *aeruginosa* CDR0031 carrying pAK1900-*waaP* or pMM-*waaP* into 6 L LB supplemented with either 100 µg/mL Cb for pAK1900-*waaP* or 50 µg/mL Cm and 1 mM IPTG for pMM-*waaP*. Cells were grown at 37 °C with shaking to OD_600_ of 1.0, harvested, and frozen at −20 °C. Cells were lysed as described in the supplementary methods. The cell lysates were centrifuged (20,000 × *g*, 60 min at 4 °C) and the supernatants were passed over a 5 mL His-Trap column. The column was washed step-wise in M buffer containing 0, 40, 80 mM imidazole, and 100 mM imidazole. WaaP-His was eluted at 5 CV M buffer containing 500 mM imidazole. Elution fractions were combined and concentrated with an Amicon Ultra-15 centrifugal filter unit MWCO 3000. The presence of WaaP-His in the elution fractions was confirmed by immunoblotting as described above. The elution fractions were further analyzed by LC-MS.

### MS conditions for protein detection

Intact protein LC-MS was performed on the WaaP-His pulldown on an Agilent 1290 UHPLC with an Agilent 6530 QToF as detector. Solvent A was 0.1% formic acid and Solvent B was 0.1% formic acid in acetonitrile. The column (PLRP-S 5 µm bead, 1000 Å pore, 2.1 mm × 50 mm, Agilent Technologies) was equilibrated and samples were loaded at 5% B and a flow rate of 0.3 mL/min at 80 °C. For elution, a gradient of 5-65% B was run over 23 min. The QToF was fitted with a Dual ESI source with a drying gas of 12 L/min at 350 °C and the nebulizer at 60 psig. Voltages were: VCap = 5500 V; Fragmentor = 175 V; Skimmer 65 V; Oct1 RF Vpp = 750 V. For data analysis, the Chemstation algorithm in MassHunter BioConfirm B.08 was used to select peaks and extract spectra which were then deconvoluted by the Maximum Entropy algorithm using the m/z range 500–2000, baseline subtraction with a baseline factor of 7, and with an output mass range of 6000–50,000 Daltons and a step of 0.5 Daltons.

### Visualization of gels and membranes, and image analysis

SDS-PAGE gels stained with Coomassie Brilliant Blue and DNA agarose gels stained with SYBR Safe were visualized using a ChemiDoc MP imaging system with Image Lab 5.2.1 software. SDS-PAGE gels stained with Sypro Orange were visualized using a Typhoon 9400 variable mode imager with Typhoon Scanner Control 5.0 and images were converted to TIFF-formatted images using ImageQuant 5.2. Immunoblotted membranes were visualized using Li-Cor Odyssey with Odyssey Infared Imaging system 3.0.30. Image analysis and processing were performed in ImageJ 1.51d^49^.

## Electronic supplementary material

Supplementary information

## Data Availability

Structure factors and refined atomic coordinates for WaaP were deposited in the RCSB Protein Data Bank under the accession number 6DFL.
