# Author Correction: Acylated-acyl carrier protein stabilizes the *Pseudomonas aeruginosa* WaaP lipopolysaccharide heptose kinase

**DOI:** 10.1038/s41598-018-34070-x

**Published:** 2018-10-23

**Authors:** Naomi N. K. Kreamer, Rajiv Chopra, Ruth E. Caughlan, Doriano Fabbro, Eric Fang, Patricia Gee, Ian Hunt, Min Li, Barbara C. Leon, Lionel Muller, Brian Vash, Angela L. Woods, Travis Stams, Charles R. Dean, Tsuyoshi Uehara

**Affiliations:** 1 Infectious Diseases, Novartis Institutes for Biomedical Research, Emeryville, CA USA; 2 Chemical Biology and Therapeutics, Novartis Institutes for Biomedical Research, Cambridge, MA USA; 3 Chemical Biology and Therapeutics, Novartis Institutes for Biomedical Research, Basel, Switzerland

Correction to: *Scientific Reports* 10.1038/s41598-018-32379-1, published online 20 September 2018

This Article contains errors in the Results section under the subheading ‘Role of acyl-ACP/WaaP interaction’.

“A similar level of WaaP-FLAG protein was produced regardless of the presence or absence of only detected in the supernatant after ultracentrifugation when acyl-ACP was present, as shown by anti-FLAG western blot (Fig. 4) and SYPRO Orange stained protein gel (Supplementary Fig. 4)”.

should read:

“A similar level of WaaP-FLAG protein was produced regardless of the presence or absence of acyl-ACP or of non-acylated ACP proteins or fatty acid in the reaction mixture (Fig. 4). However, WaaP was only detected in the supernatant after ultracentrifugation when acyl-ACP was present, as shown by anti-FLAG western blot (Fig. 4) and SYPRO Orange stained protein gel (Supplementary Fig. 4)”.

