# Supplementary material for: Acylated-acyl carrier protein stabilizes the Pseudomonas aeruginosa WaaP lipopolysaccharide heptose kinase
Source: Sci Rep. 2018 Sep 20;8:14124. doi: 10.1038/s41598-018-32379-1 (PMC6147952; doi:10.1038/s41598-018-32379-1)
Supplement: Supplementary file 1 — Supplementary information [file 41598_2018_32379_MOESM1_ESM.pdf]

Supplemental Information

Title: Acylated-acyl carrier protein stabilizes the *Pseudomonas aeruginosa* WaaP lipopolysaccharide heptose kinase

Authors: Naomi N. K. Kreamer[1], Rajiv Chopra[2]*, Ruth E. Caughlan[1], Doriano Fabbro[3], Eric Fang[1], Patricia Gee[2], Ian Hunt[2], Min Li[1], Barbara C. Leon[1], Lionel Muller[3], Brian Vash[2], Angela L. Woods[1], Travis Stams[2], Charles R. Dean[1], and Tsuyoshi Uehara[1]*

Affiliations:

[1]Infectious Diseases, Novartis Institutes for Biomedical Research, Emeryville, CA, USA

[2]Chemical Biology and Therapeutics, Novartis Institutes for Biomedical Research, Cambridge, MA, USA

[3]Chemical Biology and Therapeutics, Novartis Institutes for Biomedical Research, Basel, Switzerland

Supplementary Methods

Plasmid constructions

Plasmids (**Supplementary Table 6**) and oligonucleotide primers (**Supplementary Table 7**) used in this study are listed below. **pET21b-*waaP*** [P_{T7}*::PawaaP-his6*] and **pMM-*waaP*** [P_{lac}*::PawaaP-his6*] were constructed previously [1]. **pKOV-EcWaaP::Gm** [*pSC101-ts Cm*^{r} *sacB EcwaaP::Gm*^{r}] was constructed in several steps. The *E. coli waaP* gene was PCR amplified from an E. coli K-12 strain using primers RC113 and RC114, and cloned into pCR2.1-Topo. The DNA fragment of the generated plasmid that contained *E. coli waaP* was subcloned into pKOV utilizing BamHI and NotI digestion sites, generating pKOV-EcWaaP. Insertion into the PmlI site of pKOV-EcWaaP was a SmaI-digested fragment of the gentamycin resistance cassette (Gm^{r}) of pUCGm, generating pKOV-EcWaaP::Gm. **pETite-*waaP*-FLAG** [P_{T7}*::PawaaP-his6*] was constructed using Gibson assembly mastermix (New England Biolabs). The DNA fragment encoding *P. aeruginosa waaP*-FLAG was synthesized with codon optimized for *E. coli* (Thermo Fisher) and PCR amplified using primers NK206 and NK209. The fragment product was assembled to the backbone of pETite (Lucigen) amplified using primers NK207 and NK208 that contained 20 bp overlap sequences. **pAK1900-*waaP*** [*ori1600 Plac::waaP*$_{1\text{-}263}$*-his6*] and the plasmids expressing WaaP variants (K51A, E78A, T198A, H161A, R162A, D163A, Y165A, H168A, R191A, and R221E/R222E/R226E/R229E/R237E) were synthesized by GeneWiz. All of the other plasmids expressing WaaP variants were generated by site-directed mutagenesis of pAK1900-waaP using primers as described in **Supplementary Table 7**. **pTU448** [P_{T7}*::his6-TEV-ACP*] and **pTU450** [P_{T7}*::his6-TEV-ACP_ACPS*] were constructed using the GeneArt seamless cloning kit

(Thermo Fisher). For pTU448 construction, a DNA fragment encoding *E. coli* ACP *with* a hexahistidine tag and a cleavage site of TEV protease at the N-terminus was synthesized and used as a template for PCR using primers TU161 and TU176 to amplify a DNA product which was then cloned into the EcoRI-HindIII site of pET28a. To generate pTU450, a DNA fragment including the E. coli *acpS* gene was PCR amplified from BW25113 using TU227 and TU228, followed by stacking PCR using primers TU227 and TU229. The resultant PCR product was then cloned into the HindIII-XhoI site of pTU448, generating pTU450 which bicistronically expressed His6-TEV-ACP and AcpS with having an intergenic sequence (5' GCTTACTAGAAATAATTTTGTTTAACTTTAAGAAGGAGATATAC). **pJ414-AasS-His6** [P_{T7}*::aasS-his6*] was synthesized by ATUM with the *aasS* gene codon optimized for *E. coli* to express acyl-ACP synthetase AasS from *Vibrio harveyi* B392[2].

Expression and purification of WaaP for crystallography

E. coli BL21(DE3)/pLysS was freshly transformed with pET21b-*waaP* [P_{T7}*::PawaaP-his6*]. The fresh transformants were inoculated in SelenoMet medium base (Molecular dimensions) supplemented with 100 µg/mL carbenicillin (Cb), 34 µg/mL chloramphenicol (Cm), and 8 µg/mL methionine. After overnight incubation, the cultures were centrifuged and the pellets were resuspended in fresh medium described above with methionine replaced with selenomethionine. Cells were grown to the OD_{600} of 0.6 and cooled to 25°C prior to addition of 1 mM IPTG and grown for 4 hours at 25°C. The cells were harvested by centrifugation at 250 rpm and resuspended (5 mL per gram cell pellet) in buffer A (50 mM Tris-HCl pH 8.0, 500 mM NaCl, 50 mM arginine, 50 mM

glutamic acid, 1 mM TCEP) supplemented with 20 mM imidazole, 1 mM PMSF, and complete Protease Inhibitor Cocktail Tablets (1 tablet/50 mL - Roche Biochemicals). The cell suspension was homogenized on ice using a Polytron Mechanical Homogenizer (1x 30 sec) and then lysed with a micro-fluidizer (Microfluidics). The lysate was clarified by ultracentrifugation (138,000 x *g*, 60 min, 4°C) and passed through a batch-gravity column containing 5 mL volume equivalents of pre-washed IMAC sepharose fast flow resin (GE Healthcare). The resin was washed with 5 column volumes (CV) of lysis buffer before bound protein was eluted in 5 CV buffer A with imidazole. WaaP-His containing fractions were pooled and adjusted to 100 mM NaCl by dilution. At this stage, 1 mM EDTA added to all subsequent purification steps. WaaP-His was further purified from contaminants by ion exchange chromatography using Mono-Q and CM-sepharose columns followed by size-exclusion chromatography using a Superdex75 column (GE Healthcare). WaaP-His containing fraction were pooled and stored at 3 mg/mL in buffer A.

Crystallization and structure determination

WaaP-His in buffer (20 mM Tris-HCl pH 8.0, 100mM NaCl, 500 mM ammonium acetate, 1 mM TCEP) was mixed with equal volumes of crystallization buffer (100 mM HEPES-NaOH pH 7.4, 5% Jeffamine M-600) and crystals were grown via hanging drops at 4°C over the course of 48 hours. Crystals were flash frozen using 20% ethylene glycol as a cryo-protectant and data collected at 100K with monochromatic X-rays at a wavelength of 0.9791Å using a Dectris Pilatus 6M detector on the PXII-X10SA beamline at the Swiss Light Source, Paul Scherrer Institut, Villigen, Switzerland. Data were integrated

and scaled using the *XDS* package[3] and selenium sites identified using the SHELX software package[4]. Data diffracted to 2.5Å, albeit with a high Wilson B-factor of 69.5 $Å^2$, and a space group of $P3_121$ and unit cell dimensions of a=92.023 Å, b=92.023 Å, c=99.172Å. The electron density map generated using this information combined with solvent flattening restraints was of such quality that ~80% of the protein structure could be readily identified. Extra density and an unaccounted for selenium position led to the discovery of *E. coli* ACP protein bound to WaaP with an extended lipid attachment. Model building and refinement were carried out using COOT[5] and PHENIX[6]. Final statistics are shown in Supplementary Table 1. Atomic coordinates and structure factors are deposited in the Protein Data Bank under the accession number 6DFL.

Construction of the *E. coli waaP* deletion strain

The pKOV-EcwaaP::Gm plasmid was inserted onto the MC1061 chromosome using transformation and colonies were selected on L agar supplemented with 10 µg/mL gentamicin (Gm) and 20 µg/mL Cm. Sucrose selection was used to isolate colonies with double crossover events as described previously[7].

WaaP kinase activity assay

A radioactive filter binding assay was used to monitor the kinase reaction. The LPS substrates were prepared from *E. coli* MC1061 and the *waaP*::Gm strain as described previously[1,8]. The reaction was performed at room temperature in a final volume of 30 µL containing 20 mM Tris-HCl pH 7.5, 15 mM $MgCl_2$, 1 mM DTT. 1% DMSO, 5 µM of

ATP, 0.5 µCi ^{33}[γP]-ATP (Amersham), and LPS (300 or 30 ng/mL). Reactions started by addition of 100 or 10 nM WaaP-His and stopped after 30 min by addition of 10 µL of 4x Laemmli sample buffer and incubated at 95°C for 5 min. Fifteen microliter of each sample was loaded onto a SDS gradient gel (BioRad 4-20%) and separated by electrophoresis. The gel was dried on a Whatman paper and exposed for 72 hours to Amersham hyperfilm ECL.

Expression and purification of *E. coli* holo-ACP

E. coli His-TEV-ACP was expressed in BL21-AI without and with co-expression of *E. coli* AcpS using pTU448 and pTU450, respectively. The cells expressing His-ACP and His-TEV-ACP/AcpS were resuspended together in a 5:1 ratio in HEPES-NaOH pH 8.0 buffer containing 500 mM NaCl and 1 mM TCEP, and lysed. Complete conversion of apo-ACP to holo-ACP via addition of a 4-phosphopantetheine moiety was monitored using liquid chromatography mass spectrometry (LC-MS). His-TEV-ACP was affinity purified using a Ni-NTA column, and then cleaved by His-tagged TEV protease in 50 mM Hepes-NaOH pH 7.0, 100 mM NaCl, 1 mM TCEP. The TEV protease-treated sample containing holo-ACP was loaded onto an anion exchange column (5mL HiTrap Q HP (GELifesciences). The column was washed with 10 CV 50 mM HEPES-NaOH pH 7.0 (HiQ buffer) and 10 CV HiQ buffer containing 100mM NaCl. Holo-ACP was eluted with a 10-100% gradient over 20 CV HiQ elution buffer (50 mM HEPES-NaOH pH 7.0, 1 M NaCl). To remove remaining contaminants (mainly apo-ACP and acetylated ACP), holo-ACP was further purified using a thiopropyl sepharose 6B resin (GE Lifesciences) in TS buffer (50 mM Tris-HCl pH 7.5, 200 mM NaCl) and eluted with TE buffer (50 mM

Tris-HCl (pH 7.5), 200 mM NaCl, 20 mM DTT). Final purification was performed with HiPrep S75 Superose (GE LifeSciences) in 50 mM Tris-HCl pH 7.5 containing150 mM NaCl, 2 mM TCEP, and 10% glycerol . Holo-ACP peak fractions were pooled and concentrated with a 3K MWCO Amicon centrifugal filter (Millipore Sigma). After addition of 5 mM TCEP, the protein was stored -80°C.

Expression and purification of *V. harveyi* acyl-ACP synthase

Acyl-ACP synthase AasS from *V. harveyi* was expressed and purified from BL21-AI carrying pJ414-AasS-His6 as described above with modifications. Briefly, Protease inhibitors and universal nuclease (Thermo Fisher) were added to the cell suspension the cells were lysed by sonication. His-tagged AasS was purified using a cOmplete™ His-Tag Purification Column (Roche) with a linear gradient from 0 to 500 mM imidazole in buffer (50 mM HEPES-NaOH pH 7.5, 500 mM NaCl, 10 % glycerol, 1 mM TCEP). Fractions containing His-tagged AasS were dialyzed against AAS buffer [50 mM Tris-HCl (pH 8.0), 10% glycerol, 1 mM EDTA, 0.5 mM TCEP, and 0.002% Triton X-100] and stored at −80°C.

Acylation of holo-ACP

The acylation of holo-ACP by AasS was performed with incubation for 2 hours at room temperature (completion was monitored by MS) in a final volume of 160 µL containing 50 mM MOPS-NaOH pH 7.5, 5 mM $MgSO_4$, 5 mM ATP, 0.5 mM palmitic acid (100 mM stock in methanol), 1 mM TCEP, 0.16 mM holo-ACP, and 160 nM AasS. The buffer was

exchanged to another buffer (20 mM MOPS-NaOH pH 7.5, 500 mM NaCl, 1 mM TCEP) using a PD-10 desalting column (GE healthcare). Palmitoyl-ACP was concentrated using an Amicon Ultra-4 centrifugal filter unit MWCO 3000 (Millipore Sigma).

In vitro protein synthesis

PURExpress in vitro protein synthesis Kit (New England Biolabs) was used following the instruction with modifications. A PCR product containing a T7 promoter, codon optimized *waaP-flag*, and a T7 terminator, was amplified using pETite-*waaP*-FLAG as a template and primers NK218 and NK219. The reaction was performed at 37°C for 2.5 hours in a final volume of 100 µL containing 10 ng/uL of the PCR product (DNA template), 0.8 units/uL murine RNase inhibitor (New England Biolabs), solution A and solution B, and either no ACP, or 7.6 ng/µL apo-ACP, holo-ACP, or palmitoyl-ACP, or 200 µM palmitic acid. To separate soluble WaaP-FLAG from protein aggregates including unstable WaaP, 85 µL reaction samples containing synthesized WaaP-FLAG were ultracentrifuged at 350,000 *x g* for 15 min at 10°C in a TLA 100.2 rotor using Beckman Optima TLX Ultracentrifuge. To avoid disturbing translucent pellets, the top 60 µL in the supernatants was taken. Proteins that were synthesized in vitro were analyzed using SDS-PAGE stained with Sypro Orange and Western blotting with mouse anti-FLAG primary antibody (DYKDDDDK tag antibody, Invitrogen, MA1-91878) and IRDye 800 CW donkey anti-mouse IgG (IRDye 800CW Donkey anti-mouse, Li-cor, 926-32212).

Cell-based functional assay for WaaP variants in *P. aeruginosa* and *E. coli*

The WaaP functional assays in *P. aeruginosa* and in *E. coli* were performed using growth and EDTA MIC of *P. aeruginosa waaP*-controlled expression strain (CDR0031)[1] and novobiocin (Nov) MIC of *E. coli* Δ*waaP*[9], respectively. CDR0031 and *E. coli* Δ*waaP* were transformed with plasmids expressing WaaP wild type and variants (pAK1900-*waaP* and derivatives). Susceptibility of *P. aeruginosa* CDR0031-derived strains to EDTA was determined using a broth serial dilution assay. Strains were streaked on LB agar supplemented with 0.2% arabinose and Cb 100 µg/mL and incubated at 37°C overnight. Single colonies were used to prepare standardized cell suspension (approximately 1.5 x 10^8 colony forming units (CFU)/mL) with the BBL Prompt inoculation system (BD). The cell suspensions were diluted 1,000 fold in LB. The diluted cell suspensions were added to two-fold serial dilutions of EDTA ranging the final concentrations from 0 to 10 mM in LB in 96-well microtiter plates that were dispensed with a Janus liquid handler (Perkin Elmer). The microtiter plates were incubated overnight at 37°C and were monitored for bacterial growth with a Spectromax microtiter plate reader (Molecular Devices) at 600 nm as well as by visual observation. EDTA MIC was defined as the lowest concentration of EDTA at which less than 10% of the OD_{600} in the control well (full growth) was measured. All measurements were performed in a minimum of biological triplicate. Likewise Nov MIC for *E. coli ΔwaaP* strains expressing WaaP variants was determined using concentrations of Nov ranging from 0 to 128 µg/mL.

Immunoblotting to monitor expression levels of WaaP variants

Overnight cultures of *P. aeruginosa* CDR0031 and *E. coli ΔwaaP* carrying pAK1900-*waaP* plasmids expressing wild type WaaP-His6 and variants were inoculated in LB supplemented with 0.2% arabinose, and 100 µg/mL Cb for the *P. aeruginosa* strains or 30 µg/mL Cb for the *E. coli* strains, respectively. The fresh culture was grown at 37°C to mid-exponential phase (OD_{600} = 0.4 - 0.6). Six milliliter of the *P. aeruginosa* CDR0031 cultures and 1 mL of the *E. coli ΔwaaP* cultures were pelleted and resuspended in 50 mM MOPS-NaOH pH 7.0 to a final volume of 100 µL for *E. coli* and 120 µL for *P. aeruginosa*. An equal volume of 2x Laemmli sample buffer was added and the samples were boiled. The volumes normalized by cell density (OD_{600}) were loaded onto two 6-12% Bis-Tris SDS-PAGE gels and PAGE was run concurrently. One gel was stained with Coomassie Brilliant Blue Gels to confirm the equivalent protein levels of samples. The other gel was transferred to nitrocellulose membranes using the iBlot system (Thermo Fisher) and anti-His WaaP was detected with the iBind system (Thermo Fisher) using mouse monoclonal anti-His primary antibody (THE™ His-tag Antibody, GenScript, A00186) and IRDye 800 CW donkey anti-mouse IgG.

WaaP-His pulldown in *P. aeruginosa*

Cells were grown with 1% inoculum from overnight culture of *P. aeruginosa* CDR0031 carrying pAK1900-*waaP* or pMM-*waaP* into 6 L LB supplemented with either 100 µg/mL Cb for pAK1900-*waaP* or 50 µg/mL Cm and 1 mM IPTG for pMM-*waaP*. Cells were grown at 37°C with shaking to OD_{600} of 1.0, harvested, and frozen at -20°C. Two procedures were used to resuspend and lyse cells. One was resuspending the cell pellets in 5 mL BugBuster (Millipore Sigma) per gram of cell paste followed by cell lysis

with shaking at room temperature for 30 min. The other was resuspending the cell pellets in 40 mL M buffer (20 mM MOPS-NaOH pH 7.5, 500 mM NaCl) followed by cell lysis with the microfluidizer (Microfluidics M110-P) at 18,000 psi. During resuspension, EDTA-free Protease Inhibitor Cocktail Tablets (1 tablet/50 mL - Roche Biochemicals) was added in either procedure. The cell lysates were centrifuged (20,000 × *g*, 60 min at 4°C) to remove cell debris and the supernatants were passed over a 5 mL His-Trap column (GE Healthcare). The column was washed step-wise in M buffer containing 0, 40, and 80 mM imidazole for 5 CV and washed with 20 CV M buffer containing 100 mM imidazole. WaaP-His was eluted at 5 CV M buffer containing 500 mM imidazole. Elution fractions were combined and concentrated with an Amicon Ultra-15 centrifugal filter unit MWCO 3000 (Millipore Sigma). The presence of WaaP-His in the elution fractions was confirmed by immunoblotting as described above. The elution fractions were further analyzed by LC-MS.

MS conditions for protein detection

Intact protein LC-MS was performed on the WaaP-His pulldown on an Agilent 1290 UHPLC with an Agilent 6530 QToF as detector. Solvent A was 0.1% formic acid and Solvent B was 0.1% formic acid in acetonitrile. The column (PLRP-S 5 μm bead, 1000 Ǻ pore, 2.1 mm x 50 mm, Agilent Technologies) was equilibrated and samples were loaded at 5% B and a flow rate of 0.3 mL/min at 80°C. For elution, a gradient of 5-65% B was run over 23 min. The QToF was fitted with a Dual ESI source with a drying gas of 12 L/min at 350°C and the nebulizer at 60 psig. Voltages were: VCap = 5500V; Fragmentor = 175V; Skimmer 65V; Oct1 RF Vpp = 750V. For data analysis, the

Chemstation algorithm in MassHunter BioConfirm B.08 was used to select peaks and extract spectra which were then deconvoluted by the Maximum Entropy algorithm using the m/z range 500-2000, baseline subtraction with a baseline factor of 7, and with an output mass range of 6000-50,000 Daltons and a step of 0.5 Daltons.

Deacylation of acyl-AcpP

The WaaP-His elution was treated with 500 mM DTT overnight at room temperature to reduce the thioester bond between an acyl chain and the phophopentheine moiety of holo-AcpP. After the sample was centrifuged to remove precipitates, the supernatant was run on LC-MS as described above.

Trypsin protein digestion and MS analysis

The WaaP-His pulldown elution was diluted 20 fold with 100% ethanol and incubated overnight at -80°C. The precipitated protein was pelleted by centrifugation and resolubilized in 6 M guanidine, 50 mM Tris-HCl pH 8.0 at 95°C for 20 min. The sample was diluted 6 fold in 50 mM ammonium bicarbonate and then treated with 0.1 mg/mL sequencing grade trypsin (Promega) at 37°C for 1 hour. The sample was dried using a SpeedVac centrifuge (Eppendorf Vacufuge) and the dried material was resuspended in water. MS analysis of the peptides in the pellet was performed as described above with modifications. A Halo C18 column (2.7 µM bead core-shell, 300 Ǻ pore size, 2.1 x 150 mm, Advanced Materials Technology) was equilibrated in solvent A and the sample was loaded at 0.25 mL/min at 60°C. For elution a gradient of 5-35% solvent B was run over

50 min. The dual ESI parameters were drying gas of 10 L/min at 350°C and the nebulizer at 25 psig. Voltages are: VCap = 4000V; Fragmentor = 175V; Skimmer 65V; Oct1 RF Vpp = 750V. A data-dependent MS/MS method was run with an MS acquisition rate of 5 Hz over m/z 300-2000, and the top five precursors by abundance with charge state +2 or higher were selected for fragmentation at an acquisition rate of 3 Hz over m/z 55-2000 with a dynamic exclusion time of 6 sec. Quad isolation width was set at Medium. Collision energy was determined according to the formula CE (V) = (3.1*(m/z)/100) + 1 for +2 charge state and CE (V) = (3.6*(m/z)/100) - 4.8 for +3 and higher charge state. A Mascot generic format file was generated by Agilent MassHunter BioConfirm software version B.08 for search by Mascot MS/MS Ion database search.

Supplementary Tables:

Supplementary Table 1. Extended data table of crystallographic data and refinement statistics

**Parameters**	***P.aeruginosa* WaaP (Se-Met) complex with *E.coli* ACP**
**Space group**	$P3_121$
**Unit Cell (Å)**	a=92.023, b=92.023, c=99.172
**Resolution range (Å)**	62.1 – 2.4 (2.5 – 2.4)
**Total observations**	384311 (40060)
**Unique reflections**	19543 (1941)
**Completeness (%)**[a]	99.6 (99.6)
**Multiplicity**	19.7 (20.6)
$\langle I/\sigma(I)\rangle$[a]	32.72 (2.36)
R_{merge}[a,b]	0.065 (1.26)
$CC_{1/2}$	0.99 (0.93)
R_{cryst}/R_{free}[c]	0.227/0.272 (0.327/0.449)
**Non-H atoms**	2605
**Solvent molecules**	12
**Average *B*-factor (Å^2)**	90.32
**R.m.s.d. bond lengths (Å)**	0.007
**R.m.s.d. bond angle (°)**	0.98
**Ramachandran Plot (%)**	
**Favored**	95.39
**Allowed**	3.95
**Outliers**	0.66

[a] Numbers in parenthesis are for the highest resolution shell.

[b] $R_{merge} = \Sigma|I_h - \langle I_h\rangle|/\Sigma I_h$ over all h, where I_h is the intensity of reflection h.

[c] R_{cryst} and $R_{free} = \Sigma||F_o| - |F_c|| / \Sigma|F_o|$, where F_o and F_c are observed and calculated amplitudes, respectively. R_{free} was calculated using 5% of data excluded from the refinement.

Supplementary Table 2. WaaP variants assessed for stability and MIC* of EDTA for *P. aeruginosa* and Novobiocin for *E. coli.*

WaaP variant	Rationale	*P. aeruginosa* EDTA MIC (mM)*	MIC fold shift	Protein stability^	*E. coli* NOV MIC (µg/mL)	MIC fold shift	Protein stability ^
Wild type (WT)		10			128		
Δ*waaP* / pAK1900		ng			4		
pAK1900-WaaP-WT		5		+	64		+
K51A	ATP binding	ng	no growth	+	2	32	+
K69A**	ATP binding	NA	4		NA	4	
E78A	ATP binding	0.039	128	+	4	16	+
D188N	ATP binding	ng	no growth	+	2	32	+
T198A	phosphorylation site	5	1	+	64	1	+
H161A	HRD motif	ng	no growth	+	4	16	+
R162A	HRD motif	2.5	2	+	16	4	+
D163A	HRD motif	ng	no growth	+	4	16	+
Y165A	substrate binding	ng	no growth	+	2	32	+
H168A	substrate binding	ng	no growth	+	4	16	+
R191A	substrate binding	ng	no growth	+	4	16	+
R222A	ACP binding	0.625	8	+	32	2	+
R222E	ACP binding	2.5	2	partial	64	1	+
R226A	ACP binding	4.58	1	+	32	2	+
R226E	ACP binding	5	1	+	32	2	+
R222/226A	ACP binding	2.5	2	+	64	1	+
R222/226E	ACP binding	0.3125	16	partial	32	2	partial to +
R222/226/237E	ACP binding	0.039	128	partial	4	16	partial
R221/222/226/229/237E	ACP binding	ng	no growth	partial	4	16	partial
L143W	lipid terminus	2.5	2	partial	16	4	+
S127L	lipid terminus	2.5	2	+	32	2	+
L228A	pocket proximal to phosphopentatheine	2.5	2	+	32	2	+
L228W	pocket proximal to phosphopentatheine	5	1	+	64	1	+
L219W	mid pocket	5	1	+	32	2	+
V147L	mid pocket	0.625	8	partial	128	1	+
V147W	mid pocket	ng	no growth	-	2	32	partial to +
A214L	mid pocket	0.3125	16	partial	16	4	partial to +
A214F	mid pocket	2.5	2	+	8	8	+
A214W	mid pocket	0.3125	16	partial	4	16	partial
V147L/L219W	mid pocket	0.625	8	partial	32	2	+
V147W/214F	mid pocket	ng	no growth	-	4	16	partial

*MIC was the mode of values (a minimum of biological triplicate) defined by the minimum inhibitory concentration of chemical to inhibit 90% of growth compared to cells grown in LB in the absence of chemicals. ng: no growth.
**MICs of WT and K69A variant were measured independently in triplicates. All of these WT MICs obtained in this experiment were distinct from those obtained from other experiments. However the fold shifts in MIC by K69 were consistent in triplicates and reported. NA: not applicable.
^Protein stability was qualitatively measured by levels of WaaP-His proteins detected with Western blot shown in Supplementary Figure 2. +: stable (similar level of protein detected to WT WaaP-His). -: unstable (not detected), partial: protein bands detected but less than WT WaaP-His.

Supplementary Table 3. Trypsin digestion and MS peptide detection of ACP in the WaaP-His pulldown from CDR0031 pAK1900-*waaP.*

Protein	Peptide map
WaaP	**1** MRLVLEEPFK RLWNGRDPFE AVEALQGKVY RELEGRRTLR TEVDGRGYFV<br>**51** KIHRGIGWGE IAKNLLTAK**L PVLGAR**QEWQ AIRRLHEAGV ATMTAVAYGE<br>**101** RGSDPARQHS FIVTEELAPT VDLEVFSQDW RERPPPPRLK R**ALVEAVAR**M<br>**151** VGDMHRAGVN HRDCYICHFL LHTDKPVSAD DFR**LSVIDLH R**AQTRDATPK<br>**201** RWRNKDLAAL YFSALDIGLT RRDKLRFLRT YFRRPLREIL RDEAGLLAWM<br>**251** ERKAEKLYER KQRYGDLL
AcpP	**1** MSTIEERVKK IVAEQLGVKE EEVTNSASFV EDLGADSLDT VELVMALEEE<br>**51** FETEIPDEKA EK**ITTVQEAI DYIVAHQQ**

The amino acid sequences of *P. aeruginosa* WaaP and AcpP are shown. The peptides shown in red were detected in the WaaP-His pulldown sample treated with Trypsin.

Supplementary Table 4. MS quantitation detection of acyl-AcpP peaks in the WaaP-His pulldown from CDR0031 *pMM-waaP.*

holo(-M)-AcpP* +	Detected Mass	Theoretical Mass	Peak Area
C14:0	9160.773	9160.279	3.00E+08
Na-C14:0	9183.282	9183.268	1.00E+08
C16:1	9187.054	9186.317	1.58E+08
C16:0	9188.839	9188.333	6.46E+08
Na-C16:0	9211.226	9211.322	2.09E+08
C18:1	9214.816	9214.371	2.73E+08
C18:0	9216.445	9216.387	6.78E+07

Supplementary Table 5. Bacterial strains used or constructed in this study

Strain	Genotype or relevant characteristics	Reference or source
Escherichia coli		
Top10	*Cloning strain*	Thermo Fisher
BL21(DE3) pLysS	*F^{-} ompT $hsdS_{B}$* (r_{B}^{-} m_{B}^{-}) *gal dcm (DE3) pLysS (Cm^{r})*	Thermo Fisher
BL21-AI	*F^{-} ompT $hsdS_{B}$* (r_{B}^{-} m_{B}^{-}) *gal dcm araB::T7RNAP-tetA*	Thermo Fisher
BW25113	*F^{-} DE(araD-araB)567 lacZ4787(del)::rrnB-3, LAM^{-} rph-1 DE(rhaD-rhaB)568, hsdR514*	Baba et al[9]
MC1061	*F- hsdR2 hsdM+ hsdS+ mcrA mcrB1 araD139 delta(ara-leu)7696 delta(lacIPOZY)X74 galE15 galU galK16 rpsL thi lambda-*	ATCC 53338
waaP::Gm	MC1061 *waaP::Gm^{r}*	This study
JW3605-1	*BW25113 waaP::Km^{r}*	Baba et al[9]
Pseudomonas aeruginosa		
PAO1	*PAO1 prototroph strain K767*	DeLucia et al[1]
CDR0031	*PAO1 waaP::Gm^{r} mini-CTX(P_{ara}::waaP)*	DeLucia et al[1]

Supplementary Table 6. Plasmids used or constructed in this study

Plasmid	Genotype or relevant characteristics	Reference or source
pET21b-*waaP*	*pBR Ap^r^ P~T7~::PawaaP-his6*	DeLucia et al[1]
pKOV	*pSC101-ts Cm^r sacB*	Link et al[7]
pCR2.1-Topo	*pBR Ap^r P_{lac}::lacZα*	Thermo Fisher
pUCGM	*pUC Ap^r Gm^r(aacC1)*	Schweizer[10]
pKOV-EcwaaP::Gm	*pKOV EcwaaP::Gm^r*	This study
pMM-WaaPhis	*pMMB206(ori) Cm^r $lacI^q$ P_{lac}::PawaaP-his6*	DeLucia et al[1]
pETite-*waaP*-FLAG	*pBR Kan P_{T7}::PawaaP-flag (codon optimized for E. coli)*	This study
pJ414-AasS-His6	pBR Kan *P_{T7}::aasS-his6 (codon optimized for E. coli)*	This study
pET24a	*pBR Km^r P_{T7}::*	Novagen
pTU448	pET24a- 6His-Tev-Apo-ACP	This study
pTU450	pET24a- 6His-Tev-Apo-ACP_ACPS	This study
pAK1900	*E. coli - P. aeruginosa shuttle vector; pBR ori1600 Ap^r Cb^r P_{lac}::*	Jansons et al[11]
pAK1900-*waaP*-WT	*pAK1900 P_{lac}::PawaaP-his6*	This study
pAK1900-waaP-K51A	*pAK1900 P_{lac}::PawaaP(K51A)-his6*	This study
pAK1900-waaP-K69A	*pAK1900 P_{lac}::PawaaP(K69A)-his6*	This study
pAK1900-waaP-E78A	*pAK1900 P_{lac}::PawaaP(E78A)his6*	This study
pAK1900-waaP-D188N	*pAK1900 P_{lac}::PawaaP(D188N)-his6*	This study
pAK1900-waaP-T198A	*pAK1900 P_{lac}::PawaaP(T198A)-his6*	This study
pAK1900-waaP-H161A	*pAK1900 P_{lac}::PawaaP(H161A)-his6*	This study
pAK1900-waaP-R162A	*pAK1900 P_{lac}::PawaaP(R162A)-his6*	This study
pAK1900-waaP-D163A	*pAK1900 P_{lac}::PawaaP(D163A)-his6*	This study
pAK1900-waaP-Y165A	*pAK1900 P_{lac}::PawaaP(Y165A)-his6*	This study
pAK1900-waaP-H168A	*pAK1900 P_{lac}::PawaaP(H168A)-his6*	This study
pAK1900-waaP-R191A	*pAK1900 P_{lac}::PawaaP(R191A)-his6*	This study
pAK1900-waaP-R222A	*pAK1900 P_{lac}::PawaaP(R222A)-his6*	This study
pAK1900-waaP-R222E	*pAK1900 P_{lac}::PawaaP(R222E)-his6*	This study
pAK1900-waaP-R226A	*pAK1900 P_{lac}::PawaaP(R226A)-his6*	This study
pAK1900-waaP-R226E	*pAK1900 P_{lac}::PawaaP(R226E)-his6*	This study
pAK1900-waaP-R222/226A	*pAK1900 P_{lac}::PawaaP(R222A R226A)-his6*	This study
pAK1900-waaP-R222/226E	*pAK1900 P_{lac}::PawaaP(R222E R226E)-his6*	This study
pAK1900-waaP-R222/226/237E	*pAK1900 P_{lac}::PawaaP(R222E R226E R237E)-his6*	This study

Plasmid	Genotype or relevant characteristics	Reference or source
pAK1900-waaP-R221/222/226/229/237E	*pAK1900 P_{lac}::PawaaP(R221E/R222E/R226E/R229E/R237E)-his6*	This study
pAK1900-waaP-L143W	*pAK1900 P_{lac}::PawaaP(L143W)-his6*	This study
pAK1900-waaP-L228A	*pAK1900 P_{lac}::PawaaP(L228A)-his6*	This study
pAK1900-waaP-L228W	*pAK1900 P_{lac}::PawaaP(L228W)-his6*	This study
pAK1900-waaP-S127L	*pAK1900 P_{lac}::PawaaP(S127L)-his6*	This study
pAK1900-waaP-L219W	*pAK1900 P_{lac}::PawaaP(L219W)-his6*	This study
pAK1900-waaP-V147L	*pAK1900 P_{lac}::PawaaP-(V147L)-his6*	This study
pAK1900-waaP-V147W	*pAK1900 P_{lac}::PawaaP(V147W)-his6*	This study
pAK1900-waaP-A214L	*pAK1900 P_{lac}::PawaaP(A214L)-his6*	This study
pAK1900-waaP-A214F	*pAK1900 P_{lac}::PawaaP(A214F)-his6*	This study
pAK1900-waaP-A214W	*pAK1900 P_{lac}::PawaaP(A214W)-his6*	This study
pAK1900-waaP-V147L/L219W	*pAK1900 P_{lac}::PawaaP(V147L/L219W)-his6*	This study
pAK1900-waaP-V147W/A214F	*pAK1900 P_{lac}::PawaaP(V147W/A214F)-his6*	This study

Supplementary Table 7. Oligonucleotides used in this study

Primer name	Description	Sequence (5'-3')
NK159	WaaP_D188N_F	CTCGGTGATCAACCTGCACCGTGC
NK160	WaaP_D188N_R	AGGCGGAAATCGTCCGCG
NK161	WaaP_R222A_F	ACTGACGCGTGCCGACAAGCTACG
NK162	WaaP_R222A_R	CCGATGTCCAGCGCAGAG
NK163	WaaP_R222E_F	ACTGACGCGTGAAGACAAGCTACGCTTC
NK164	WaaP_R222E_R	CCGATGTCCAGCGCAGAG
NK165	WaaP_R226A_F	CGACAAGCTAGCGTTCCTGCGCACCTATTTCC
NK166	WaaP_R226A_R	CGACGCGTCAGTCCGATG
NK167	WaaP_R226E_F	CGACAAGCTAGAATTCCTGCGCACCTATTTCC
NK168	WaaP_R226E_R	CGACGCGTCAGTCCGATG
NK169	WaaP_R222A/R226A_F	GCTAGCGTTCCTGCGCACCTATTTC
NK170	WaaP_R222A/R226A_R	TTGTCGGCACGCGTCAGTCCGATGTC
NK171	WaaP_R222E/R226E_F	GCTAGAATTCCTGCGCACCTATTTC
NK172	WaaP_R222E/R226E_R	TTGTCTTCACGCGTCAGTCCGATGTC
NK173	WaaP_L219W_F	GGACATCGGATGGACGCGTCGCG
NK174	WaaP_L219W_R	AGCGCAGAGAAATACAATG
NK175	WaaP_L228W_F	GCTACGCTTCTGGCGCACCTATTTCC
NK176	WaaP_L228W_R	TTGTCGCGACGCGTCAGT
NK177	WaaP_L228A_F	GCTACGCTTCATGCGCACCTATTT
NK178	WaaP_L228A_R	TTGTCGCGACGCGTCAGT
NK179	WaaP_S127L_F	CGAGGTGTTCCTGCAGGACTGGCGCGAACGTCC
NK180	WaaP_S127L_R	AGGTCCACGGTCGGCGCC
NK181	WaaP_V147W_F	GGTCGAGGCGTGGGCGCGGATGGTCG
NK182	WaaP_V147W_R	AGCGCGCGCTTGAGCCGC
NK183	WaaP_A214F_F	GTATTTCTCTTTCCTGGACATCGGACTGAC
NK184	WaaP_A214F_R	AATGCCGCCAGATCCTTG
NK185	WaaP_L143W_F	CAAGCGCGCGTGGGTCGAGGCGGTGG
NK186	WaaP_L143W_R	AGCCGCGGCGGTGGAGGA
NK194	WaaP_A214W_F	GTATTTCTCTTGGCTGGACATCGGAC
NK195	WaaP_A214W_R	AATGCCGCCAGATCCTTG
NK196	WaaP_A214L_F	GTATTTCTCTCTGCTGGACATCGGAC
NK197	WaaP_A214L_R	AATGCCGCCAGATCCTTG
NK198	WaaP_V147L_F	GGTCGAGGCGCTGGCGCGGATGG

NK199	WaaP_V147L_R	AGCGCGCGCTTGAGCCGC
NK220	WaaP_K69A_F	GCTCACCGCCGCCCTCCCGGTGC
NK221	WaaP_K69A_R	AGGTTCTTGGCGATCTCG
NK214	codon optimized WaaP-FLAG for insert_f	TATAAGAAGGAGATATACATATGCGTCTGGTTCTGGAAGA
NK215	codon optimized WaaP-FLAG pETite backbone_R	TCTTCCAGAACCAGACGCATATGTATATCTCCTTCTTATA
NK206	codon optimized WaaP-FLAG pETite backbone_R	TATAAGAAGGAGATATACATATGAGGCTGGTGCTGGAAGA
NK207	codon optimized WaaP-FLAG for insert_F	TCTTCCAGCACCAGCCTCATATGTATATCTCCTTCTTATA
NK208	codon optimized WaaP-FLAG for insert_R	AAGATGATGATGATAAATAATAGAGCGGCCGCCACCGCTG
NK209	codon optimized WaaP-FLAG pETite backbone_f	CAGCGGTGGCGGCCGCTCTATTATTTATCATCATCATCTT
NK218	F peTite PURexpress	GGCACCTAATACGACTCACTATAGGG
NK219	R peTite PURexpress	CCGGATATAGTTCCTCCTTTCAGC
RC113	E.coli waaP for 2	CCAGCCATGCATTATCCATCCCTAT
RC114	E.coli waaP rev 2	ACGTAAAGCGTTAACTCAGTCGCCA
TU161	His Tev Stacking F3 for	GAATTGTGAGCGGATAACAATTCCGAATTCAGGAGGTAAAACATATGCAC
TU176	EcApoAcp pET24GA Rev	GCTCGAGTGCGGCCGCAAGCTTACGCCTGGTGGCCGTTGA
TU227	ACP-S R	GTGGTGGTGGTGCTCGAGTGCGGCCGCTTAACTTTCAATAATTACCG
TU228	ACP-S F4	AGAAATAATTTTGTTTAACTTTAAGAAGGAGATATACATGGCAATATTAGGTTTAGGC
TU229	ACP-S F5	CGGCCACCAGGCGTAAGCTTACTAGAAATAATTTTGTTTAACT

Supplemental Figures:

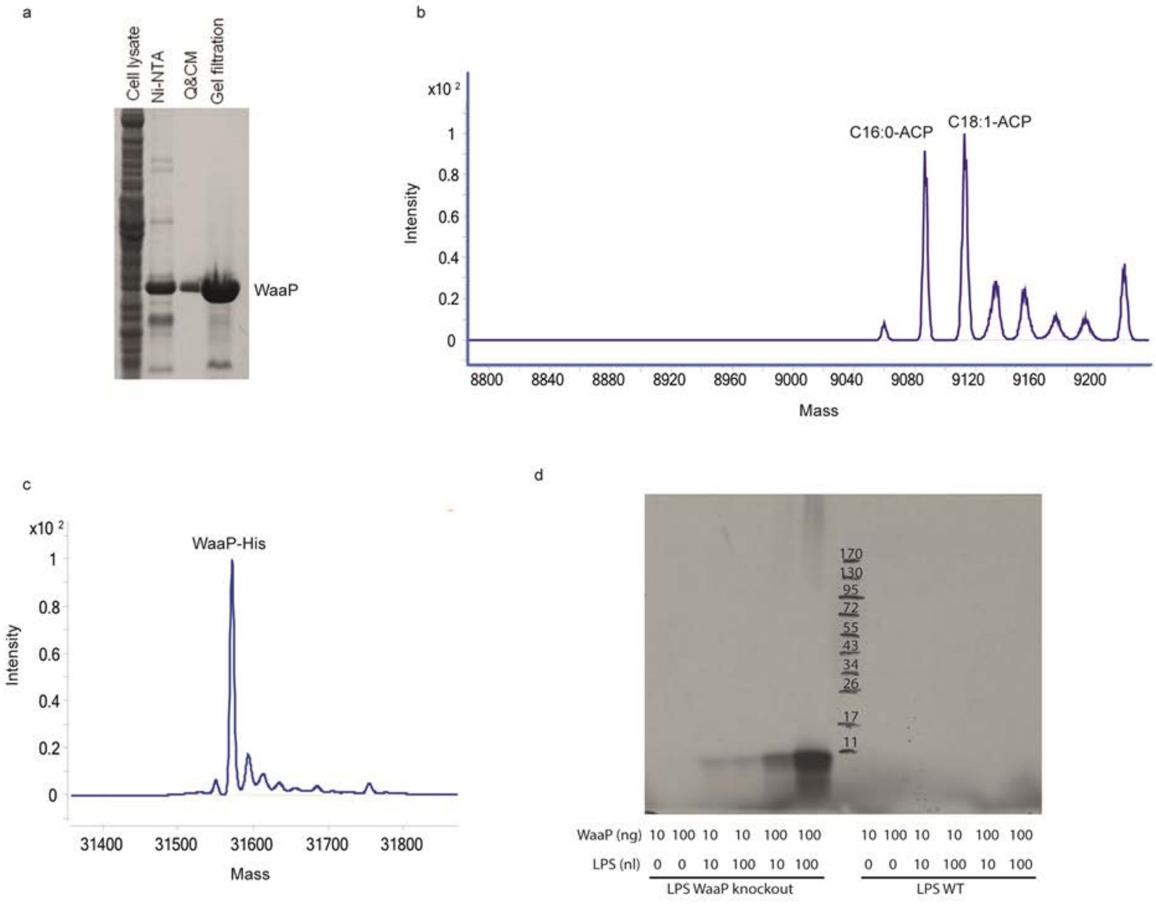

Supplementary Figure 1: Purification, MS identification of WaaP and acyl-ACP, and kinase activity for WaaP-His protein used in crystallography

(**a**) Increasing enrichment of WaaP after each column purification step is shown in SDS-PAGE. The protein fraction after gel filtration purification was used to generate the crystal structure shown in Fig. 1a, (**b,c**) analyzed by mass spectrometry, and (**d**) used in WaaP kinase assay. (**b**) The MS trace in mass regions of acyl-ACP shows peaks with two major masses were consistent with C16:0-ACP and C18:1-ACP from *E. coli*. (**c**) The MS peak of WaaP-His matched the expected molecular weight (31,572). (**d**) Radioactive detection of phosphotransferase activity of WaaP using radiolabeled [γ-^{33}P]-ATP and LPS from either WaaP knockout *E. coli* MC1061 cells (left side of gel) or MC1061 cells (right side of gel) in SDS-PAGE. WaaP was active specifically on its substrate, unphosphorylated LPS (black spots on left side of gel), but not on the fully phosphorylated LPS.

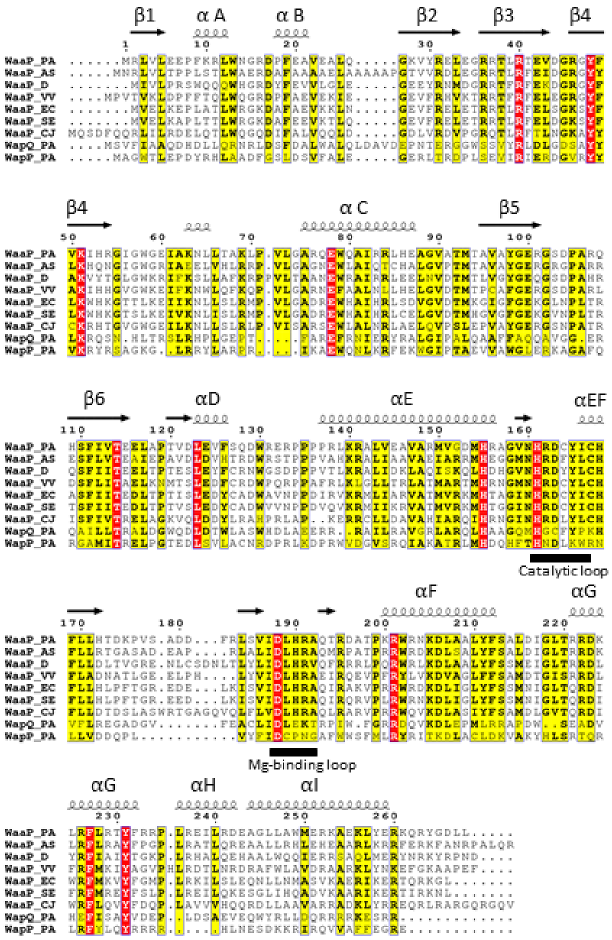

Supplementary Figure 2. Primary sequence alignment of WaaP homologues and closely related LPS heptose kinases.

WaaP homologues from diverse set of organisms, where PA = *P. aeruginosa* PAO1, AS = *Azoarcus* sp. BH72, D = *Desulfuromonas acetoxicans*, VV = *Victivallis vadensis*, EC = *E. coli* K12 MG1655, SE = *Salmonella enterica* RSK2980, and CJ = *Cellvibrio japonicus.* Features of the enzyme are annotated including secondary structural elements are shown above the sequences (beta sheets represented by arrow and alpha helices represented by loops) and key catalytic domains. Residues highlighted in red are conserved for all sequences and those highlighted in yellow are similar.

a

Stability of WaaP variants in *P. aeruginosa* CDR0031

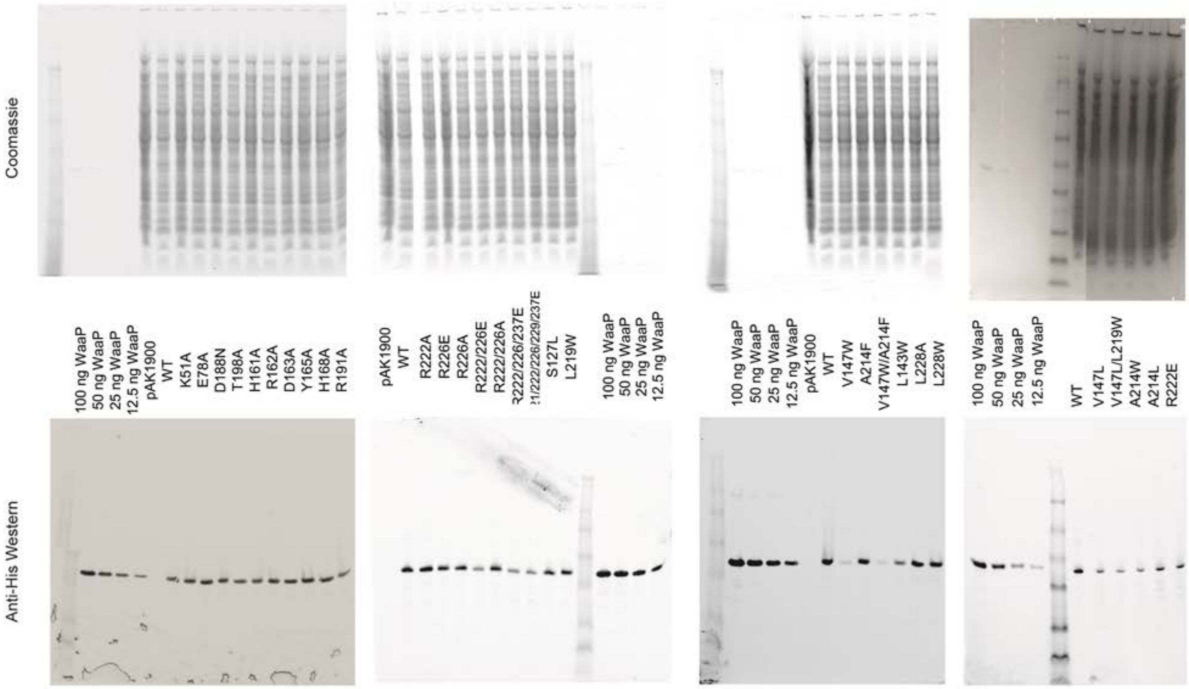

b

Stability of WaaP variants in *E. coli* Δ*waaP*

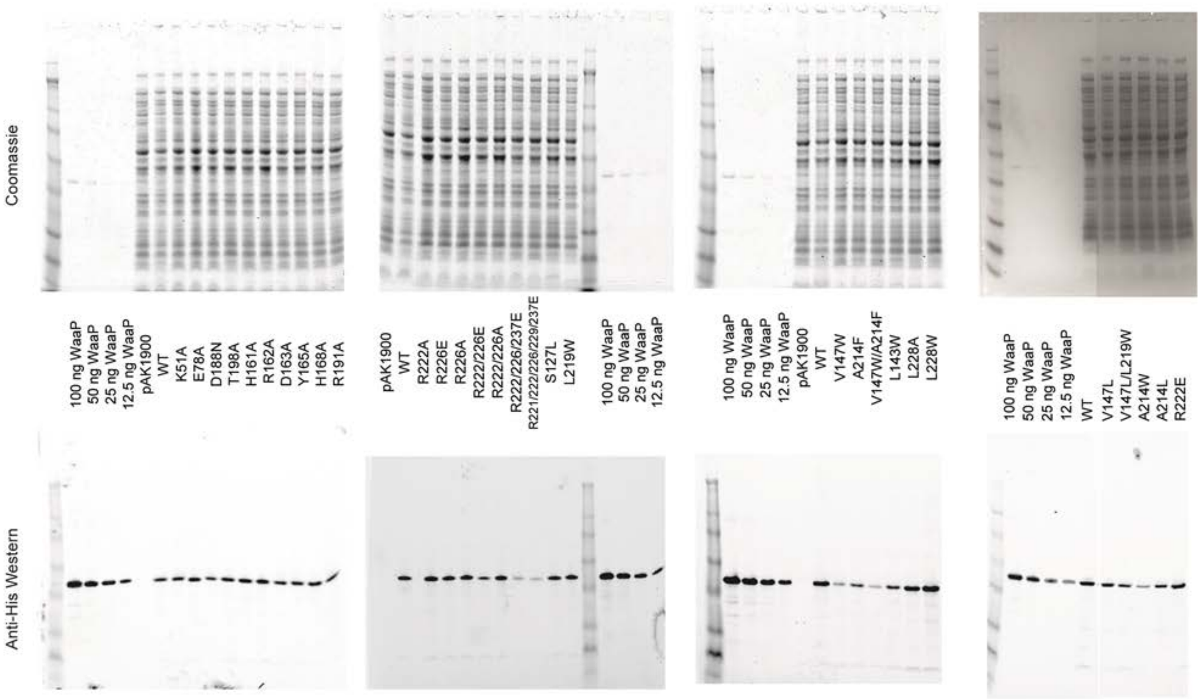

Supplementary Figure 3. Stability of WaaP variants expressed in cells.

Protein levels of WaaP variants expressed in exponentially growing cells of *P. aeruginosa* CDR0031 (**a**) and *E. coli* Δ*waaP* (**b**). Cells were collected and subjected to SDS-PAGE and anti-His western blot analysis, where loading amounts were normalized to OD_{600}. SDS-PAGE verified that the approximately same amounts of total proteins were loaded onto each western blot. Cells expressing wild type WaaP-His and purified WaaP-His protein (12.5, 25, 50, and 100 ng) were loaded for comparative controls. Gels shown are representatives of biological duplicates. These results are summarized in Fig 2b, 4b, 5b and Supplementary Table 2.

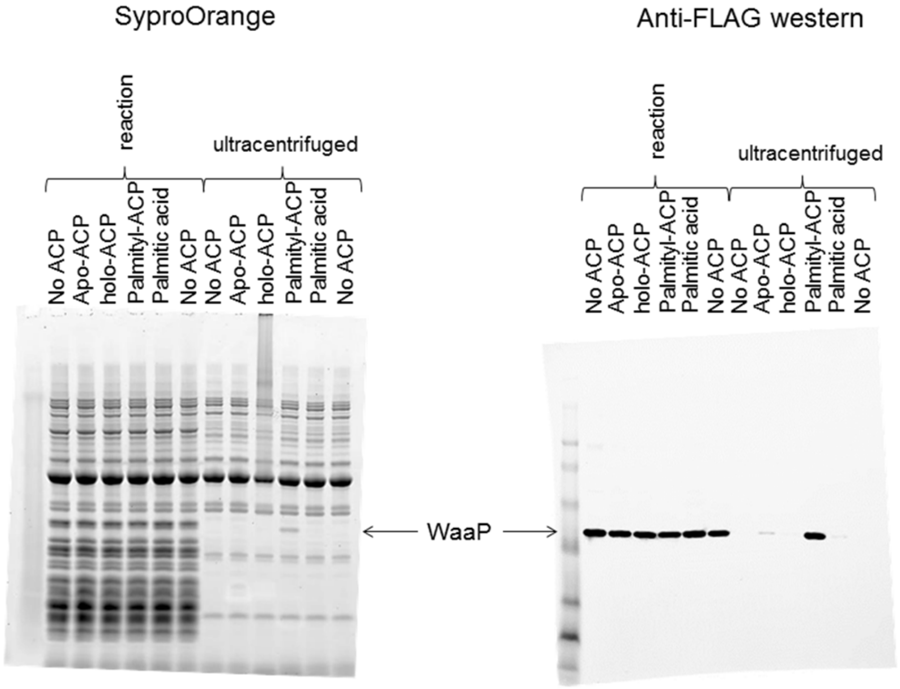

Supplementary Figure 4. In vitro synthesis of soluble WaaP only in the presence of acyl-ACP.

WaaP-FLAG was expressed from a DNA fragment containing P_{T7}::*waaP*-FLAG using an in vitro transcription and translation system in the absence of ACP or in the presence of apo-ACP (unmodified ACP), holo-ACP (phosphopentheinylated ACP), palmitoyl-ACP, or free palmitic acid. Sypro Orange stained protein gel (left) and the uncropped immunoblot with anti-FLAG antibody (right) are shown. WaaP was produced in every reaction sample at similar level (left side of both gels). After ultracentrifugation, WaaP was only present in the supernatant when expressed in the presence of acyl-ACP (right side of both gels).

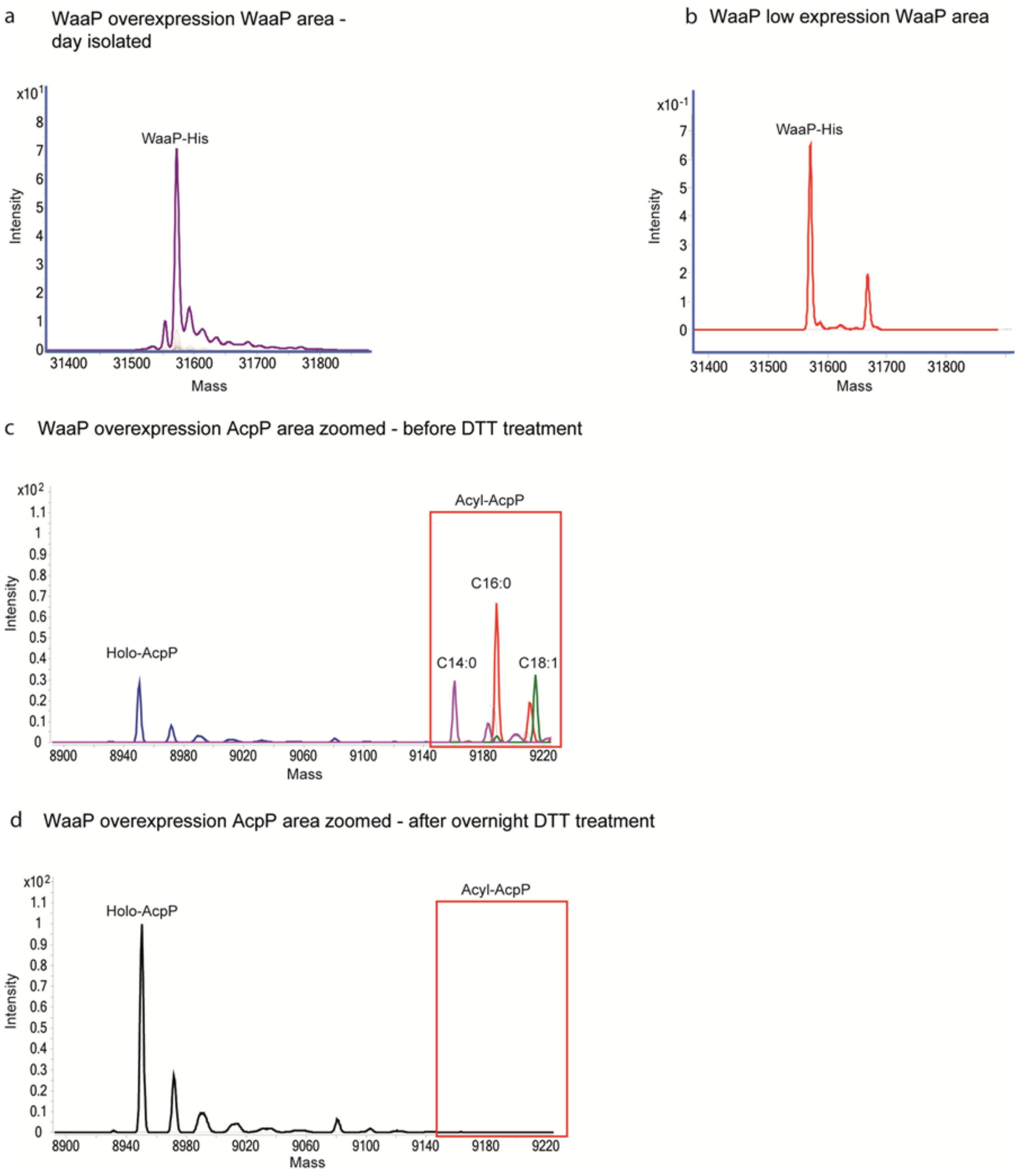

Supplementary Figure 5. Mass spectra of WaaP-His pulldowns from *P. aeruginosa.*

(**a,b**) Detection of WaaP-His peaks with a mass of 31,572 in the pulldown samples from CDR0031 carrying pMM-*waaP* (**a**) and from CDR0031 carrying pAK1900-*waaP* (**b**),

showing lower expression from pAK1900-*waaP*. (**c,d**) Confirmation of acyl-AcpP peaks using thioester cleavage of the acyl chain by reduction with DTT. Peaks in the 9140-9220 mass range in the WaaP-His pull down sample from CDR0031 carrying pMM-*waaP* before (**c**) and after treatment of DTT (**d**) were shown. The trace in **d** shows a loss of acyl-AcpP species with a concomitant increase in holo-AcpP after overnight incubation with DTT.

References:

1. DeLucia, A.M. et al. Lipopolysaccharide (LPS) Inner-Core Phosphates Are Required for Complete LPS Synthesis and Transport to the Outer Membrane in Pseudomonas aeruginosa PAO1. *mBio* **2**(2011).
2. Jiang, Y., Chan, C.H. & Cronan, J.E. The soluble acyl-acyl carrier protein synthetase of Vibrio harveyi B392 is a member of the medium chain acyl-CoA synthetase family. *Biochemistry* **45**, 10008-19 (2006).
3. Kabsch, W. XDS. *Acta Crystallographica Section D: Biological Crystallography* **66**, 125-132 (2010).
4. Sheldrick, G.M. Macromolecular phasing with SHELXE. *Z. Kristallogr.* **217**, 644-650 (2002).
5. Emsley, P., Lohkamp, B., Scott, W.G. & Cowtan, K. Features and development of Coot. *Acta Crystallogr D Biol Crystallogr* **66**, 486-501 (2010).
6. Adams, P.D. et al. PHENIX: a comprehensive Python-based system for macromolecular structure solution. *Acta Crystallographica Section D: Biological Crystallography* **66**, 213-221 (2010).
7. Link, A.J., Phillips, D. & Church, G.M. Methods for generating precise deletions and insertions in the genome of wild-type Escherichia coli: application to open reading frame characterization. *J Bacteriol* **179**, 6228-37 (1997).
8. Darveau, R.P. & Hancock, R.E. Procedure for isolation of bacterial lipopolysaccharides from both smooth and rough Pseudomonas aeruginosa and Salmonella typhimurium strains. *Journal of Bacteriology* **155**, 831-838 (1983).
9. Baba, T. et al. Construction of Escherichia coli K-12 in-frame, single-gene knockout mutants: the Keio collection. *Molecular Systems Biology* **2**, 2006.0008-2006.0008 (2006).
10. Schweizer, H.D. Small broad-host-range gentamycin resistance gene cassettes for site-specific insertion and deletion mutagenesis. *Biotechniques* **15**, 831-4 (1993).
11. Jansons, I. et al. Deletion and transposon mutagenesis and sequence analysis of the pRO1600 OriR region found in the broad-host-range plasmids of the pQF series. *Plasmid* **31**, 265-74 (1994).
